# STIM1 promotes migration, phagosomal maturation and antigen cross-presentation in dendritic cells

**DOI:** 10.1038/s41467-017-01600-6

**Published:** 2017-11-24

**Authors:** Paula Nunes-Hasler, Sophia Maschalidi, Carla Lippens, Cyril Castelbou, Samuel Bouvet, Daniele Guido, Flavien Bermont, Esen Y. Bassoy, Nicolas Page, Doron Merkler, Stéphanie Hugues, Denis Martinvalet, Bénédicte Manoury, Nicolas Demaurex

**Affiliations:** 1 Department of Cell Physiology and Metabolism, University of Geneva, Geneva, 1211 Switzerland; 2 Laboratory of Normal and Pathological Homeostasis of the Immune System, INSERM UMR1163, Paris, 75015 France; 3 Université Paris Descartes, Sorbonne Paris Cité, Faculté de médecine Paris Descartes, Paris, 75015 France; 4 Department of Pathology and Immunology, University of Geneva, Geneva, 1211 Switzerland; 5 Division of Clinical Pathology, Geneva University Hospital, Geneva, 1211 Switzerland; 6 Institut National de la Santé et de la Recherche Médicale, Unité 1151, Paris, 75014 France; 7 Centre National de la Recherche Scientifique, Unité 8253, Paris, 75014 France

## Abstract

Antigen cross-presentation by dendritic cells (DC) stimulates cytotoxic T cell activation to promote immunity to intracellular pathogens, viruses and cancer. Phagocytosed antigens generate potent T cell responses, but the signalling and trafficking pathways regulating their cross-presentation are unclear. Here, we show that ablation of the store-operated-Ca^2+^-entry regulator STIM1 in mouse myeloid cells impairs cross-presentation and DC migration in vivo and in vitro. *Stim1* ablation reduces Ca^2+^ signals, cross-presentation, and chemotaxis in mouse bone-marrow-derived DCs without altering cell differentiation, maturation or phagocytic capacity. Phagosomal pH homoeostasis and ROS production are unaffected by STIM1 deficiency, but phagosomal proteolysis and leucyl aminopeptidase activity, IRAP recruitment, as well as fusion of phagosomes with endosomes and lysosomes are all impaired. These data suggest that STIM1-dependent Ca^2+^ signalling promotes the delivery of endolysosomal enzymes to phagosomes to enable efficient cross-presentation.

## Abstract (web summary)

STIM proteins sense Ca^2+^ depletion in the ER and activate store-operated Ca^2+^-entry (SOCE) in response, a process associated with dendritic cell functions. Here the authors show STIM1 is the major isoform controlling SOCE in mouse dendritic cells and provide a mechanism for its requirement in antigen cross-presentation.

## Introduction

Dendritic cells (DC) are phagocytic immune cells that link innate and adaptive immunity by processing and presenting ingested antigens. One of the unique functions of DCs is cross-presentation, which is a specific type of antigen presentation that occurs via major histocompatibility complex class I (MHC-I) molecules to activate CD8^+^ T cells and help generate antigen-specific immunity to intracellular pathogens, viruses and cancer cells. Cross-presentation of antigens acquired through phagocytosis produces more potent T cell responses than soluble antigens^1^, and DCs are particularly involved in phagocytosis and transport of large particles (> 500 nm) to draining lymph nodes^2^. However, the precise molecular mechanisms by which cross-presentation of phagocytosed antigens occurs are not well understood. Cross-presentation requires a number of proteins normally located in the endoplasmic reticulum (ER), such as tapasin, calreticulin, ERp57 and the translocon Sec61^1^. DC phagosomes are particularly rich in ER proteins^3, 4^, but the signalling and trafficking mechanisms regulating the relationship between the ER and the phagosome during cross-presentation is controversial^3, 5–8^.

Ca^2+^ signalling is linked to a variety of DC functions including differentiation, maturation, migration, cytokine secretion, phagocytic ingestion and antigen presentation^9^. However, most studies have relied on the use of non-specific inhibitors, ionophores and chelators, which can have pleiotropic effects. Stromal interaction molecule (STIM) proteins, which include the two isoforms STIM1 and STIM2 each with multiple splice variants, are ER transmembrane proteins that sense the ER Ca^2+^ depletion resulting from activation of inositol trisphosphate (InsP_3_) receptors^10^. They subsequently remodel the ER and promote the formation and expansion of membrane contact sites (MCS) between the ER and plasma membrane (ER–PM MCS), where they directly activate PM-resident Ca^2+^ channels of the ORAI and transient receptor potential (TRPC) families in the process termed store-operated Ca^2+^-entry (SOCE)^11^. Electrophysiological studies suggest that SOCE is the major Ca^2+^ entry pathway in DCs^12^, and one study suggests that STIM2 is the major isoform regulating DC function in mice^13^. In human peripheral blood monocyte-derived DCs genetic manipulation of ORAI1 and STIM1 suggested that STIM1 is critical for DC maturation^14^, but another study suggests that STIM1 and STIM2 are dispensable for a variety of DC functions in mice^15^.

Although the classic model of cross-presentation postulates that antigens are first partially proteolysed in phagosomes, retrotranslocated from the phagosome to the cytosol where they are further processed by the proteasome, and then reimported into the ER for loading onto ER-resident MHC-I molecules^1^, some studies propose that non-canonical trafficking pathways involving fusion of ERGIC vesicles and recycling endosomes with phagosomes may explain the presence of ER proteins on phagosomes^7, 8, 16^. However, the signalling and targeting mechanisms that control these pathways are unclear. In neutrophils, we previously showed that STIM1 promotes the formation of contact sites between the ER and phagosomes that allow localized Ca^2+^ signalling^17^, raising the question of whether STIM1 may also affect the association between phagosomes and the ER in DCs.

In the present study, we characterize the consequences of genetic ablation of *Stim1* on DC functions including differentiation, maturation, migration, phagocytosis and cross-presentation. Our data establish that STIM1 is the major isoform controlling SOCE in mouse DCs and suggest that STIM1 promotes cross-presentation at least in part by increasing Ca^2+^-dependent migration. In addition, STIM1 promotes the formation of contact sites between the ER and phagosomes that in turn produce localized Ca^2+^ signals that may potentiate proteolysis and fusion of phagosomes with endosomes and lysosomes.

## Results

### *Stim1* promotes cross-presentation of phagocytosed antigens

**Fig. 1:**
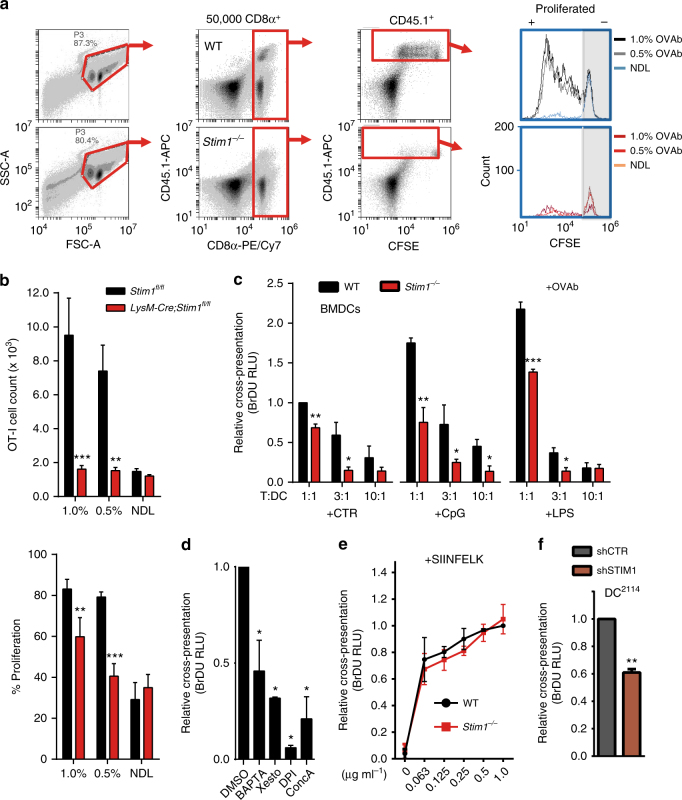
STIM1 promotes cross-presentation in vivo and in vitro. **a** Representative flow cytometry gating strategy of cells isolated from draining (DL) and non-draining (NDL) lymph nodes of mice injected with 1.0 or 0.5% OVA-coated beads (OVAb), and with 1 × 10^6^ CD45.1^+^ OT-I cells. Out of 50,000 CD8α^+^ cells, the total number of CD45.1^+^ OT-I cells and their corresponding CFSE fluorescence were analysed. **b** OT-I cells were strongly decreased in DL but not NDL of CD45.2^+^ *LysM-Cre; Stim1* ^*fl/fl*^ mice as compared to *Stim1* ^*fl/fl*^ littermates (upper graph). Similarly, OT-I proliferation, as assessed by CFSE dilution, was significantly decreased in STIM1-deficient mice (lower graph). *N* = 3 pairs of mice. **c** Cross-presentation of OVA antigens to OT-I cells by BMDCs exposed to OVAb (4 h) was quantified as the BrDU incorporation after 72 h of co-culture. *Stim1* ablation reduced cross-presentation in vitro, both in immature BMDCs and in cells matured with 1 μg mL^−1^ LPS or 0.1 μM CpG, at varying OT-I:BMDC (T:DC) ratios. *N* = 3, in triplicate wells. Ratio 1:1 T:DC in unstimulated WT cells = 1. **d** Pre-incubation with 40 μM BAPTA-AM or 1 μM Xesto reduced cross-presentation, similar to known inhibitors 0.2 nM ConcA or 10 μM DPI, applied as positive controls. *N* = 3, in triplicate wells. Control DMSO condition = 1. **e** Incubation with varying doses of the OVA(257–264) fragment SIINFEKL (1 h) induced similar levels of cross-presentation in WT and STIM1-deficient BMDCs. *N* = 3, in triplicate wells. **f** Reduced cross-presentation was also observed in DC^2114^ + shSTIM1, as compared to control (+shCTR) as assessed with BrDU. Phagocytic targets added at 20:1 targets:cells. *N* = 3, in triplicate wells. Error bars are means ± SEM, p* < 0.5, **p < 0.1, ***p < 0.01 using a two-way ANOVA and Sidak’s post test for **b**, **c** and **e**, and a Student’s *t*-test for **d** and **f**

To determine whether STIM1 promotes cross-presentation, PBS solutions with 0, 0.5, or 1% ovalbumin (OVA)-coated beads (OVAb) were injected into footpads of *LysM-Cre*; *Stim1* ^*fl/fl*^ mice bearing the CD45.2 allele and a conditional deletion of the *Stim1* gene in myeloid cells, and into control CD45.2 *Stim1* ^*fl/fl*^ littermates. After 24 h, CD45.1, H2-K^b^/OVA(257–264)-reactive CD8α^+^ T cells (OT-I) labelled with carboxyfluorescein succinimidyl ester (CFSE) were injected retro-orbitally. Draining (DL) and non-draining (NDL) lymph nodes were harvested after 72 h, and the total number of CD45.1^+^ OT-I cells within the CD8α^+^ population, as well as the CFSE dilution as a measure of OT-I proliferation, were determined. The full gating strategy is shown in Fig. 1a. STIM1 deficiency dramatically reduced the total number of CD45.1^+^ OT-I cells within the CD8α^+^ gate in DL of mice injected with 1 or 0.5% OVAb but not in NDL (Fig. 1a, b) or in lymph nodes from mice injected with PBS (Supplementary Fig. 1a). OT-I proliferation was reduced in a dose-dependent manner (Fig. 1a, b), indicating that effectively, cross-presentation was impaired upon *Stim1* ablation in myeloid cells.

We next examined whether cell-specific DC cross-presentation was impaired by co-culturing bone-marrow-derived DCs (BMDC) pulsed with OVAb along with OT-I cells in vitro and measuring OT-I proliferation after 72 h by bromodeoxyuridine (BrdU) incorporation. Western blots confirmed that STIM1 protein expression was reduced by > 90% in BMDCs isolated from *LysM-Cre*; *Stim1* ^*fl/fl*^ mice (labelled henceforth *Stim1* ^*−/−*^ for simplicity) as compared to BMDCs isolated from *Stim1* ^*fl/fl*^ mice (labelled henceforth WT, Supplementary Fig. 1b). Maturation with microbial products CpG oligodeoxynucleotides and lipopolysaccharide (LPS) increased OT-I proliferation by ∼twofold (Fig. 1c), while Ca^2+^ chelation with BAPTA-AM or inhibition of InsP_3_-receptors with Xestospongin C (Xesto) decreased cross-presentation, as did the inhibitors of the NADPH oxidase and the vacuolar-ATPase, diphenylpropidium iodide (DPI) and concanamycin A (ConcA) respectively, applied as positive controls (Fig. 1d). This indicates that cross-presentation is Ca^2+^-dependent. *Stim1* ablation reduced the relative levels of cross-presentation to similar extents in immature and mature cells, an effect that was most pronounced at intermediate DC:T cell ratios (Fig. 1c). Similar results were obtained when measuring OT-I proliferation by CFSE dilution (Supplementary Fig. 1c, d). The *Stim1*-dependent defect in cross-presentation was specific for processing of phagocytosed antigens, as STIM1-deficient BMDCs were equally efficient at cross-presenting as WT when pulsed with varying doses of SIINFEKL peptide, an OVA(257–264) fragment that does not require proteolytic processing (Fig. 1e, Supplementary Fig. 1e). To confirm that abrogating STIM1 expression reduces cross-presentation in vitro, a CD8α^+^ DC cell line highly efficient at cross-presentation, DC^2114^ ^18^, was transduced with either control or shRNA directed against STIM1 (shCTR and shSTIM1 respectively). STIM1 depletion was verified by Western blot (Supplementary Fig. 1b). Similar to primary BMDCs, cross-presentation was reduced by STIM1 depletion in DC^2114^ cells (Fig. 1f). DC^2114^ induced nearly fourfold higher levels of OT-I proliferation as compared to BMDCs (Supplementary Fig. 1f), generating large colonies easily discerned by microscopy where the effect of STIM1 knockdown was clearly apparent (Supplementary Fig. 1g). These data indicate that STIM1 promotes the cross-presentation of phagocytosed antigens in vivo and that this effect is recapitulated in vitro in primary or cultured DCs.

### *Stim1* ablation impairs the Ca^2+^-dependent migration of BMDCs

**Fig. 2:**
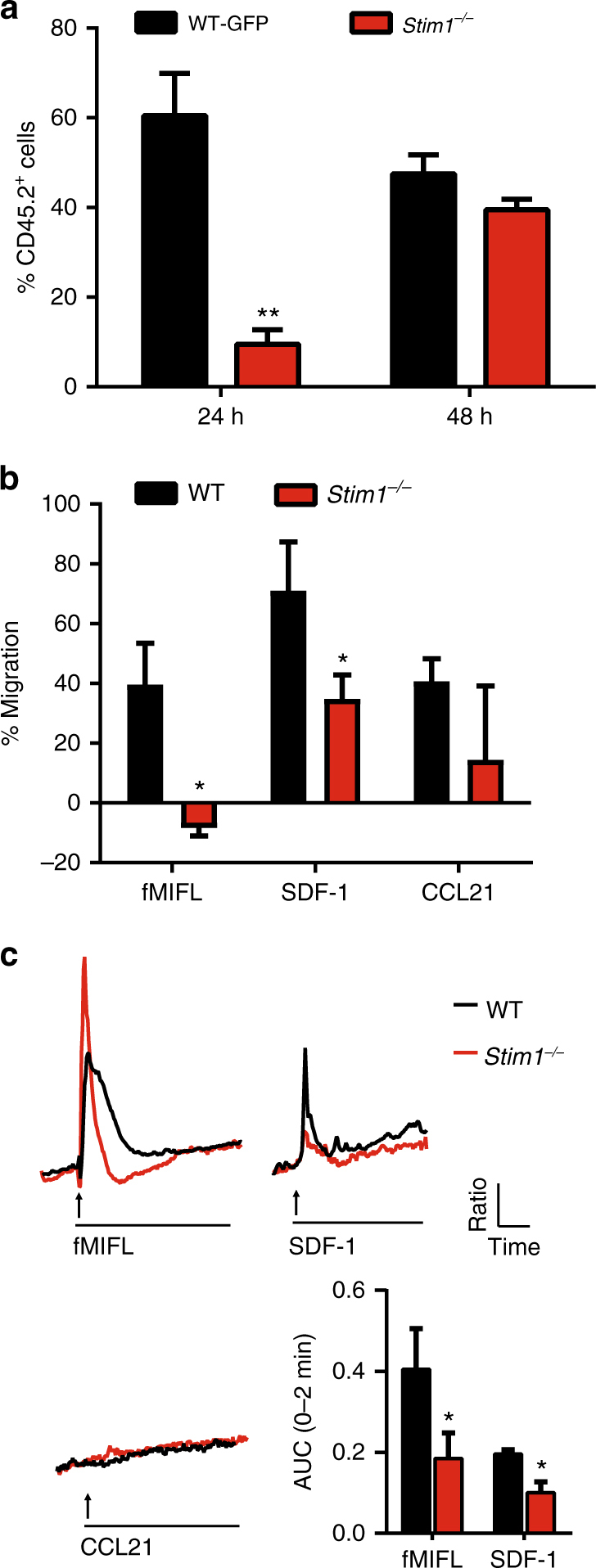
STIM1 promotes Ca^2+^-dependent migration in vivo and in vitro. **a** CD45.1 congenic wild-type hosts were injected with a mixture of 1 × 10^6^ wild-type GFP+ (WT-GFP), 1 × 10^6^ *Stim1* ^*−/−*^ BMDCs and 0.5% OVAb. DL were isolated and the relative percentage of WT:*Stim1* ^*−/−*^ (CD11c^+^, CD45.2^+^) cells analysed by flow cytometry. Full gating strategy is shown in Supplementary Fig. 2a. The proportion of *Stim1* ^*−/−*^ cells was significantly reduced 24 h but not 48 h after injection. *N* = 4 pairs of mice. **b** A 24 h transwell migration assay revealed 40–65% increases in migration towards fMIFL, SDF-1 and CCL21 chemoattractants in WT cells. *Stim1* ^*−/−*^ BMDC showed reduced migration towards fMIFL and SDF-1 but not CCL21 (all applied at 10^−6^M) as compared to WT. Full gating strategy is shown in Supplementary Fig. 2b. *N* = 3 in triplicate wells. **c** Ca^2+^ transients induced by acute exposure (arrows) of chemoattractants, as measured using Fura-2 (traces representative average of 14–22 cells). Quantification of the area under the curve (AUC) of the first 2 min after chemoattractant addition (right bottom panel) revealed significantly lower Ca^2+^ entry in *Stim1* ^*−/−*^ BMDCs in response to fMIFL and SDF-1, while no Ca^2+^ entry in response to CCL21 was detected for either genotype. *N* = 4 (coverslips each) containing: 68/66/72 (WT) or 47/56/52 (*Stim1* ^*−/−*^) cells for fMIFL/SDF-1/CCL21. Error bars are means + SEM, *p < 0.5, **p < 0.1 using a two-way ANOVA and Sidak’s post test for **a** and a Student’s *t*-test for **b** and **c**

The decrease in the total number of OT-I cells in the DL of *STIM1*-deficient mice, a measure of functional cross-presentation, could result from defective migration of DCs to and from the site of OVAb injection where antigens are captured, to the lymph nodes where DCs encounter OT-I cells. To test whether DC migration to lymph nodes was impaired in vivo, a 1:1 mixture of BMDCs from CD45.2 WT mice expressing cytosolic GFP (WT-GFP) and CD45.2 *Stim1* ^*−/−*^ BMDCs was co-injected into footpads of CD45.1 host mice together with 0.5% OVAb. DLs were collected at 24 and 48 h post injection, and the CD45.1^−^CD45.2^+^ population analysed for GFP expression. The percentage of GFP-expressing WT cells in DLs was strongly and significantly higher at 24 h post injection, whereas the initial 1:1 ratio was nearly recovered at 48 h (Fig. 2a, Supplementary Fig 2a). BMDC migration was next examined in vitro using transwell chambers and the chemoattractant fMIFL to mimic microbial peptides found at the site of infection, SDF-1 (also known as CXCL12), a chemokine expressed by lymphatic vessels^19^, or CCL21 (also known as Exodus-2 or SLC), the major chemokine eliciting DC homing to lymph nodes (Fig. 2b, Supplementary Fig. 2b). No differences between baseline levels of migration between WT and *Stim1* ^*−/−*^ cells were observed, and surface expression of the SDF-1 and CCL21 receptors CXCR4 and CCR7 was similar in WT and *Stim1* ^*−/−*^ BMDCs matured or not with CpG, LPS, or OVAb (Supplementary Fig. 2c, d). WT cells showed strong, moderate and no Ca^2+ ^transients evoked in response to fMIFL, SDF-1, and CCL21, respectively. STIM1-deficient cells displayed a brief Ca^2+^ elevation characteristic of ER-Ca^2+^ release by InsP_3_ –generating receptors, with an impaired delayed elevation characteristic of SOCE, resulting in a ∼50% reduction of the integrated Ca^2+^ response (Fig. 2c). Interestingly, the size of the migration defect was congruent with the size of the induced Ca^2+^ transients evoked by the same concentration of each chemoattractant (10^−6^ M) in STIM1-deficient cells, with fMIFL evoking the largest transients and the greatest migration defect, while CCL21 recruited STIM-deficient cells effectively without evoking Ca^2+^ responses (Fig. 2b, c). Preserved CCL21 signalling explains the delayed migration of STIM1-deficient DCs to lymph nodes and suggests that the CCL21-evoked Ca^2+^ transients reported in DCs^20^ might depend on other factors such as prostaglandins^21^. Together, these results imply that impaired Ca^2+^-dependent DC migration contributes to defective functional cross-presentation in vivo by delaying the recruitment of DCs to the site of injection and their subsequent rapid migration to lymph nodes. However, other factors are likely to contribute, since DCs eventually accumulate in lymph nodes.

### *Stim1* ablation impairs global and localized Ca^2+^ signals

**Fig. 3:**
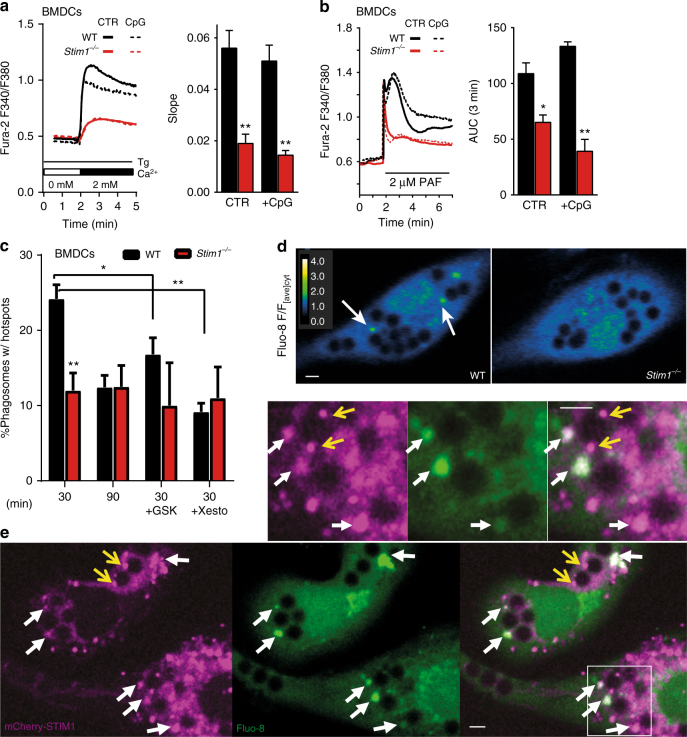
STIM1 localizes near phagosomes and promotes global and local Ca^2+^ signals in BMDCs. **a**, **b** *Stim1* ablation strongly decreased SOCE in immature and CpG-matured BMDCs, measured as the slope of Ca^2+^-re-entry after store depletion with 1 μM Tg in Ca^2+^-free medium and 2 mM Ca^2+^ re-addition **a**, or as the area under the curve (AUC) of the first 3 min after acute exposure to the agonist PAF (2 μM, **b**). Traces are averages of 11–23 cells. *N* = 10/6; 3/4 (coverslips each) containing 129/119; 26/70 (WT) or 139/98; 67/97 (*Stim1* ^*−/−*^) cells for Tg: CTR/CpG; PAF: CTR/CpG. **c**, **d** Localized Ca^2+^ signalling near phagosomes (green dots, arrows, **d**) was measured in BMDCs loaded with 4 μM Fluo-8 and 2.5 μM BAPTA after 30 min or 90 min of exposure to targets, and in the absence or presence of 10 μM GSK or 1 μM Xesto after 30 min (**c**). *Stim1* ablation reduced periphagosomal Ca^2+^ hotspots at 30 but not 90 min, and both GSK and Xesto reduced hotspot frequency in WT but not *Stim1* ^*−/−*^ cells (**c**). *N* = 5/4;7/4;5/3;6/4 coverslips representing 870/794; 1916/1154; 898/814; 1124/1372 phagosomes; 196/178; 370/238; 270/265; 375/211 cells for WT/*Stim1* ^*−/−*^ 30;90:30+GSK;30+Xesto. The colour-coded ratio images are Fluo-8 fluorescence/average cytosolic Fluo-8 fluorescence and show representative hotspots (**d**). **e** BMDCs transduced with mCherry-STIM1 (magenta) and loaded with Fluo-8 (green) as above display periphagosomal accumulations of STIM1 fluorescence that co-localize with Ca^2+^ hotspots (white arrows), as well as puncta that show no Ca^2+^ activity (yellow arrows). 20:1 target:cell ratio. Bars = 3 μm. Error bars are means + SEM, *p < 0.5, **p < 0.1 using a Student’s *t*-test

**Fig. 4:**
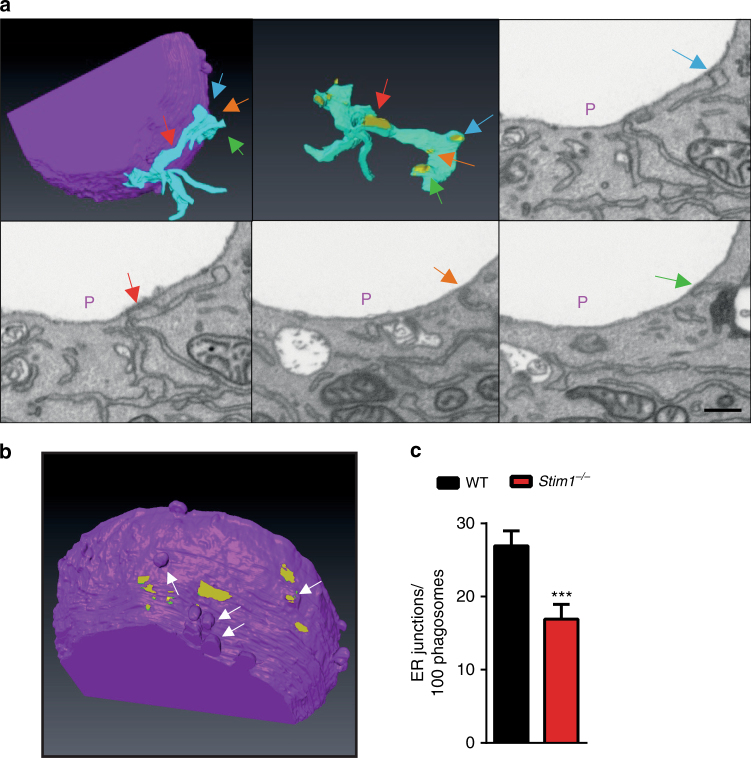
STIM1 promotes ER–Phagosome membrane contact sites. **a** 3D reconstruction (top left and middle) of FIB-SEM images (top right and bottom) shows that contiguous ER membranes (light blue) make multiple contact sites (coloured arrows and yellow highlight) with a single phagosome (purple). **b** The vicinity of contact sites (yellow highlight) is associated with an increased occurrence of bulges in the phagosome surface away from the beads, reminiscent of vesicular fission or fusion activity (arrows). **c** Quantification of FIB-SEM slices revealed that *Stim1* ablation reduced the frequency of ER–Ph MCS (also called ER junctions). *N* = 55/26 slices representing a total of 170/35 phagosome cross-sections in WT/*Stim1* ^*−/−*^ BMDCs respectively. OVAb added at 20:1 targets:cells, cells fixed 30 min after exposure to targets. Bar = 100 nm. Error bars are means + SEM, *p < 0.5, **p < 0.1 using a Student’s *t*-test

SOCE can be functionally measured in isolated cells as the rate of Ca^2+^ entry after internal Ca^2+^stores are depleted in the absence of external Ca^2+^. To further characterize the Ca^2+^ signalling defects of STIM1-deficient DCs, we used the SERCA inhibitor thapsigargin (Tg) to passively deplete ER Ca^2+^ stores and assess global SOCE. *Stim1* ablation reduced SOCE by ∼70% in both immature and mature BMDCs (Fig. 3a), a defect that could not be accounted for by changes in ORAI1 or STIM2 expression (Supplementary Fig. 3a). STIM1 knockdown also reduced SOCE by 70% in DC^2114^ cells (Supplementary Fig. 3b), and Ca^2+^ responses evoked by platelet activating factor (PAF) in BMDCs were markedly impaired regardless of maturation (Fig. 3b, Supplementary Fig. 3c). In addition to global Ca^2+^ signals, localized Ca^2+^ hotspots occur during phagocytosis in macrophages, neutrophils and phagocytic fibroblasts^17, 22^. In fibroblasts, these signals coincide in time and space with the STIM1-mediated tethering of ER cisternae to phagosomes, reflecting both ER Ca^2+^ release as well as the opening of phagosomal Ca^2+^ channels^17^. We therefore checked whether periphagosomal Ca^2+^ hotspots and ER–phagosome MCS (ER–Ph MCS) were also present in DCs. Indeed, periphagosomal Ca^2+^ hotspots were observed in BMDCs exposed to OVAb for 30 min and were reduced by ∼50% upon *Stim1* ablation (Fig. 3c, d). Periphagosomal Ca^2+^ hotspots were observed even after 90 min of OVAb exposure, although their frequency was reduced and their occurrence was no longer STIM1 dependent (Fig. 3c). SOCE channel inhibition with GSK7975A (GSK, 10 µM) added concomitantly with OVAb, and InsP_3_R inhibition with Xesto (1 µM) 20 min after OVAb addition, reduced hotspot frequencies in WT but not STIM1-deficient cells (Fig. 3c). Although Xesto may affect STIM1 recruitment to phagosomes by preventing ER store depletion, these data suggest that both Ca^2+^ release from phagosomes as well as Ca^2+^ release from recruited ER stores contribute to local Ca^2+^ signals. In addition, in transduced BMDCs, periphagosomal mCherry-STIM1 puncta co-localized with periphagosomal hotspots (Fig. 3e, white arrows). Periphagosomal STIM1 puncta that were not associated with Ca^2+^ hotspots were also observed (Fig. 3e, yellow arrows), but the majority of hotspots co-localized with STIM1 clusters. Immunostainings confirmed that STIM1 puncta were observed near phagosomes (Supplementary Fig. 4a). Similar to neutrophils, ER cisternae closely apposed (<30 nm) to phagosomes were observed in BMDCs exposed to OVAb for 30 min, as visualized using both classic transmission electron microscopy (Supplementary Fig. 4b) as well as focused-ion-beam scanning electron microscopy (FIB-SEM) (Fig. 4a–c). 3D reconstruction of FIB-SEM stacks confirmed that sites of ER–phagosome contact represented sections contiguous with the bulk ER (Fig. 4a). Interestingly, the 3D reconstruction also revealed that the same ER cisterna may display multiple sites of contact with the same phagosome (highlighted in yellow in Fig. 4a and Supplementary Movie 1). Additionally, whereas the phagosomal membrane closely follows the surface of the ingested beads for most of the phagosomal surface, in certain instances the region surrounding these ER–Ph MCS displayed multiple sites of bulging away from the bead that were reminiscent of vesicular trafficking (fusion or fission) activity (Fig. 4b, arrows). Quantification of EM slices revealed that similar to neutrophils^17^, *Stim1* ablation significantly reduced the number of ER–Ph MCS in BMDCs (Fig. 4c). Together, these data imply that STIM1 depletion has a major impact on global SOCE in DCs, as well as on localized Ca^2+^ signals mediated through ER–Ph MCS.

### *Stim1* is dispensable for DC maturation and phagocytosis

**Fig. 5:**
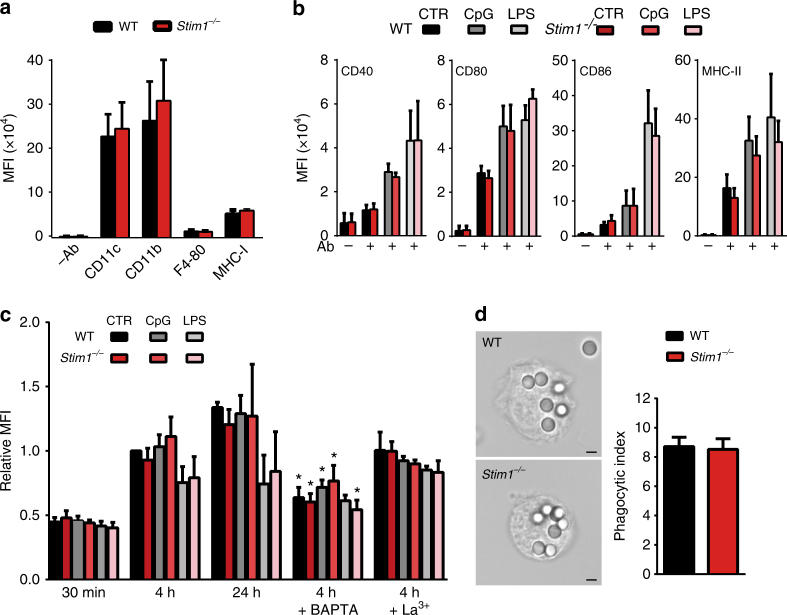
*Stim1* ablation does not affect BMDC differentiation, maturation, or phagocytic rate. **a** BMDCs express CD11c, CD11b, low levels of F4/80 and MHC-I to similar extents regardless of STIM1 expression. *N* = 8/5/5 for CD11c/CD11b/F4/80. Values of cellular autofluorescence (−Ab) are shown for comparison. The gating strategy is shown in Supplementary Fig. 5a. **b** Expression of CD40, CD80, CD86 and MHC-II in immature cells or in cells matured with either CpG or LPS was similar in cells expressing or not STIM1. The full gating strategy is shown in Supplementary Fig. 2c. *N* = 7/5/3 for CTR/CpG/LPS for CD40/CD80, 8/5/7 for CD86 and 8/5/5 for MHC-II. **c** Phagocytosis of YG- Fluoresbrite-OVA-coated beads (OVAb) was decreased by loading cells with 40 μM BAPTA but not by exposing cells to the non-specific SOCE blocker LaCl_3_ (50 μM), and was similar in immature and mature STIM1-deficient cells as compared to cells from wild-type littermates. *N* = 4/5/3/3/3 for 30 min/4 h/24 h/BAPTA/La^3+^. The full gating strategy is shown in Supplementary Fig. 2c. **d** The absence of phagocytic defect in STIM1-deficient cells was confirmed by quantifying phagocytosis by microscopy. Phagocytic targets added at 20:1 targets:cells. *N* = 3. MFI = mean fluorescence intensity. Black bar = 3 μm. Error bars are means + SEM, *p < 0.5 using a Student’s *t*-test

Previous reports suggest that DC differentiation and maturation are dependent on Ca^2+^ signalling^22^. BMDCs differentiated from bone-marrow precursors expressed high levels of CD11c, CD11b and low levels of F4/80, characteristic of conventional-like DCs^23^ (Fig. 5a, Supplementary Fig. 5a). In both wild-type and *Stim1* ^*−/−*^ BMDC cultures, > 90% of cells expressed CD11c and expression of all three markers were similar in both genotypes, as was surface levels of MHC-I, indicating that DC differentiation was unaffected by *Stim1* ablation (Fig. 5a). DC^2114^ cells were CD8α^+^, CD11b^−^, F4/80^−^ and B220^−^, (characteristic of CD8^+^ lymphoid-like DCs^23^), and expressed similar levels of CD11c when transduced with either shCTR or shSTIM1 (Supplementary Fig. 5b, c). Exposure of BMDCs to TLR ligands induces DC maturation characterized in part by an upregulation of cell-surface markers such as CD40, CD80, CD86 and MHC-II. When BMDCs were exposed to maturation stimuli CpG or LPS for 18 h the cell-surface expression of CD40, CD80, CD86 and MHC-II was similarly increased in both genotypes (Fig. 5b, Supplementary Fig. 2c). STIM1-silenced DC^2114^ cells were also equally capable of upregulating maturation markers in response to CpG (Supplementary Fig. 5d). We then checked whether acute administration of these TLR ligands evoked Ca^2+^ responses in immature cells. LPS evoked small Ca^2+^ transients in ∼30% of BMDCs and these signals were of lower amplitude in *Stim1* ^*−/−*^ cells, while CpG did not induce any detectable Ca^2+^ transient in either genotype (Supplementary Fig. 5e).

In neutrophils phagocytic ingestion is Ca^2+^- and STIM1-dependent^17, 24, 25^, and phagocytosis is Ca^2+^-dependent in DCs^9, 15^. Phagocytic ingestion was therefore quantified by exposing BMDCs to OVA-coated Fluoresbrite-YG beads for 30 min, 4 h and 24 h. While Ca^2+^ chelation by pre-incubation of cells with BAPTA-AM reduced phagocytosis by ∼40% in control WT cells, inhibition was less pronounced or not significant in mature cells, and neither *Stim1* ablation nor the addition of the non-specific SOCE inhibitor La^3+^ significantly affected phagocytosis in either immature or mature BMDCs (Fig. 5c, Supplementary Fig. 2c). Furthermore, phagocytosis was unaltered in STIM1-deficient cells as assessed by microscopy (Fig. 5d). Together these data suggest that defects in DC differentiation, maturation or rates of phagocytic ingestion are unlikely to contribute significantly to the impaired cross-presentation of STIM1-deficient DCs.

### *Stim1* ablation does not alter phagosomal ROS or pH

**Fig. 6:**
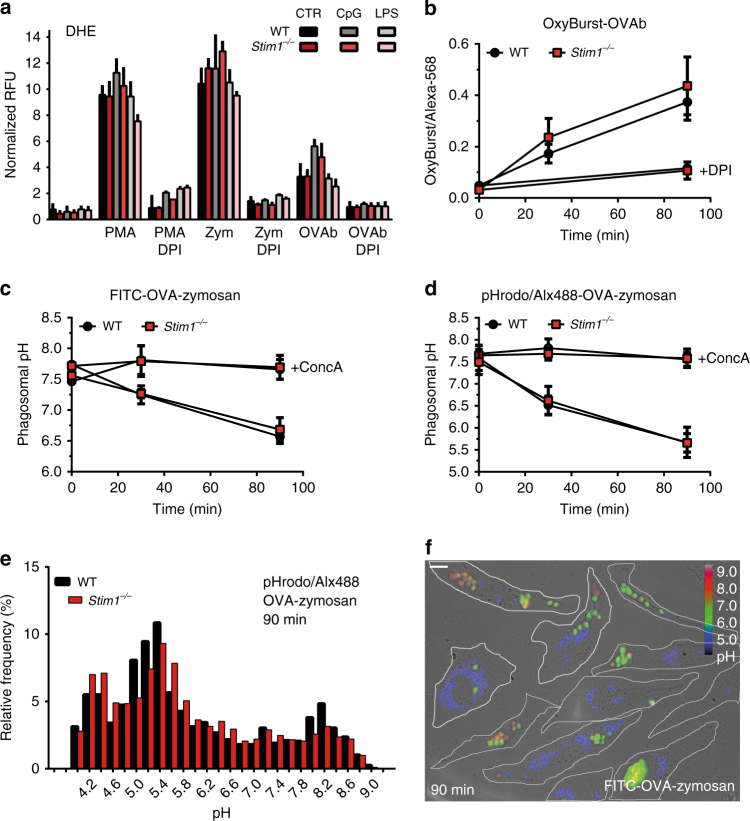
*Stim1* ablation does not affect ROS production or phagosomal pH. **a** Intracellular ROS production was measured in immature and CpG or LPS-matured BMDCs loaded with 30 μM DHE, and exposed to OVA-coated beads (OVAb), zymosan (Zym) or 100 nM PMA. *N* = 4, triplicate wells. **b** ROS production during phagocytosis (30 and 90 min) was assessed by exposing BMDCs to OVAb coupled to OxyBurst and Alexa-568. In **a** and **b**, DPI (10 μM) blocked the DHE or OxyBurst signal, but no differences were detected upon *Stim1* ablation. *N* = 6/4/4 coverslips containing 84/54/58 (WT) or 85/50/53 (*Stim1* ^*−/−*^) cells, for 30 min/90 min/DPI. **c**, **d** Phagosomal acidification (30 and 90 min) was measured using ratiometric or pseudo-ratiometric imaging by exposing cells to FITC-coupled (**c**) and pHrodo/Alexa-488-coupled (**d**) OVA-coated zymosan, respectively. Phagosomal acidification was blocked by ConcA (0.2 nM), but there were no differences upon *Stim1* ablation. *N* = 3 coverslips for all conditions, comprising 3218/2826/1713/3533 (WT) or 2722/5295/2608/4438 (*Stim1* ^*−/−*^) FITC-phagosomes, and 2612/2194/700/746 (WT) or 1740/2218/670/862 (*Stim1* ^*−/−*^) pHrodo phagosomes, for 30 min/90 min/30 min ConcA/90 min ConcA. **e** Histogram of all combined 90 min pHrodo phagosomal pH values for WT and *Stim1* ^*−/−*^ BMDCs shows the broad and bimodal distribution of phagosomal pH in these cells. **f** Measurements of phagosomal pH by microscopy revealed the heterogeneity of the pH of individual phagosomes even within the same cell. Cells are outlined in white, the image is a merge of the brightfield and 480/440 colour-coded ratio channels 90 min after addition of FITC-OVA-zymosan. pH values obtained from calibration curves (Supplementary Fig. 6b) are matched to the ratio colour-coded bar. Phagocytic targets added at 20:1 targets:cells. White bar = 10 μm. Error bars are means ± SEM

Several phagosomal maturation steps such as actin shedding, ROS production and fusion with granules or lysosomes are Ca^2+^-dependent in neutrophils and macrophages^9, 22^. In DCs, lower phagosomal proteolysis, higher phagosomal ROS and reduced phagosomal acidity correlate with higher cross-presentation^26^. In this context, reduced phagosomal ROS production, or increased acidity or proteolytic activity might lead to impaired cross-presentation in STIM1-deficient DCs. We therefore measured intracellular ROS production with dihydroethidium (DHE) in cells exposed to OVAb or zymosan particles (phagocytic targets known to elicit high levels of ROS), with the NADPH oxidase activator phorbol myristate acid (PMA) and inhibitor DPI serving as positive and negative controls. Intracellular ROS levels increased by 10–12-fold upon PMA or zymosan stimulation and by 4−6-fold upon exposure to OVAb, showing the highest level in cells matured with CpG (Fig. 6a). Extracellular ROS levels, measured with Amplex Rex, increased by 4–6-fold upon PMA exposure, whereas OVAb did not produce detectable extracellular ROS (Supplementary Fig. 6a). *Stim1* ablation had no significant effect on ROS production in any condition tested (Fig. 6a, Supplementary Fig. 6a). BMDCs were also exposed to OVAb coupled to OxyBurst and Alexa-568, and imaged at 30 min and 90 min after bead addition. The ratio of OxyBurst to Alexa-568 fluorescence increased progressively following particle ingestion and was inhibited by DPI, yet again, no significant differences were detected (Fig. 6b).

We next examined whether phagosomal pH might be affected by *Stim1* ablation. Phagosomal pH was measured by exposing BMDCs to either FITC-coupled or to pHrodo-Red- and Alexa-488-coupled zymosan. Zymosan was used instead of OVAb as FITC and pHrodo coupling to amine-functionalized beads was unstable. BMDCs were imaged after 30 and 90 min of exposure to zymosan, and pre-treatment with ConcA used to document V-ATPase-mediated acidification. Phagosomal pH was determined from in situ calibration curves (Supplementary Fig. 6b) and measurements with FITC and pHrodo were made in parallel, using cells originating from the same mice. BMDCs showed a broad distribution of phagosomal pH ranging from very acidic to very basic (∼pH 4–9), displaying a roughly bimodal distribution, with an average pH that steadily decreased over the course of the recordings in a ConcA-sensitive manner (Fig. 6c–e). Phagosomes within the same cell had widely differing pH values, indicating that heterogeneity is determined at the level of single phagosomes (Fig. 6f). Regardless of the dye used, the phagosomes of *Stim1* ^*−/−*^ BMDCs displayed an average pH similar to WT cells (6.57 ± 0.11 vs. 6.68 ± 0.19 and 5.67 ± 0.34 vs. 5.66 ± 0.21 after 90 min for WT and *Stim1* ^*−/−*^ cells using FITC and pHrodo respectively, Fig. 6c, d). Endosomal pH was additionally assessed by loading cells with Alexa-568 and FITC-coupled dextran. Although a trend for a more alkaline endosomal pH in *Stim1* ^*−/−*^ cells was apparent, the difference was not significant (Supplementary Fig. 6c).

### *Stim1* ablation impairs phagosomal proteolysis and fusion

**Fig. 7:**
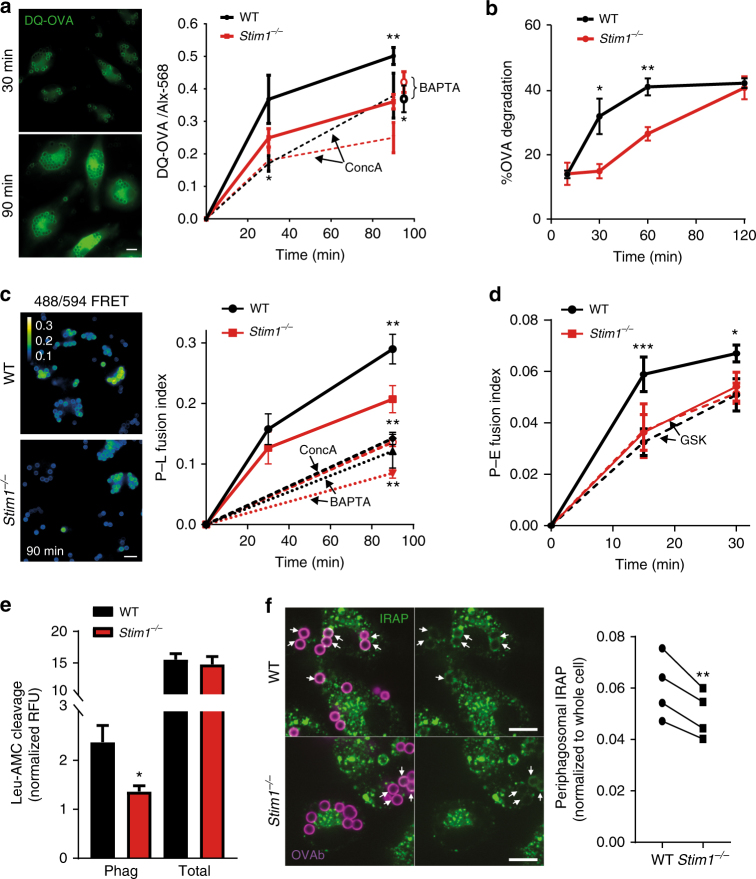
STIM1 promotes phagosomal proteolysis and endomembrane fusion. **a** Proteolysis was measured in BMDCs exposed to DQ-OVA-Alexa-568 beads. STIM1-deficient cells showed lower levels of DQ-OVA fluorescence at 90 min (right panel). Pre-incubation with BAPTA-AM (40 μM) reduced WT proteolysis to levels similar to STIM1-deficient cells. Lines representing the BAPTA condition are omitted and 90 min points are displaced to the right for clarity. ConcA (0.2 nM) only partially inhibited proteolysis. *N* = 5/10/3/5/3 (coverslips) comprising a total of 236/1050/237/432/200 (WT) or 264/975/218/405/217 (*Stim1* ^*−/−*^) cells for 30/90/30 + ConcA/90 + ConcA/90 + BAPTA(min). **b** OVA degradation was measured with anti-OVA immunostainings of isolated phagosomes by flow cytometry. Values are % OVA degradation. Full gating strategy is shown in Supplementary Fig. 7a. Proteolysis was decreased at 30 and 60 min after ingestion. *N* = 3. **c** Phago–lysosome (P–L) fusion was measured by exposing Alexa-488-OVAb to cells loaded with lysosomal FRET acceptor Alexa-594-HA. Colour-coded images (left) show the FRET signal at 90 min for WT and STIM1-deficient cells. P–L fusion indices are matched to the colour-coded bar. P–L fusion was decreased in STIM1-deficient cells as compared to WT at 90 min, whereas BAPTA-AM loading further decreased P–L fusion and eliminated differences between WT and *Stim1* ^*−/−*^ cells. ConcA decreased P–L fusion to similar levels as BAPTA-loaded cells. *N* = 3/4/4/3 (coverslips) comprising 470/1009/1691/797 (WT) or 643/1314/1617/503 (*Stim1* ^*−/−*^) phagosomes for 30/90/90 + ConcA/90 + BAPTA (min). **d** Phago–endosome (P–E) fusion was measured by exposing Alexa-488-OVAb to cells loaded with endosomal FRET acceptor Alexa-594-dextran. P–E fusion was decreased in STIM1-deficient cells as compared to WT. Addition of GSK (10 μM) eliminated differences in P–E fusion. *N* = 3 (coverslips) comprising a total of 213/244/144/146 (WT) or 210/154/233/253 (*Stim1* ^*−/−*^) cells for 15/30/15 + GSK/30 + GSK(min). Phagocytic targets added at 20:1. **e** Leu-AMC fluorescent substrate cleavage was reduced in phagosomes isolated from STIM1-deficient cells but not in whole-cell lysates, *N* = 3. **f** Periphagosomal IRAP, quantified from single confocal slices, was reduced in STIM1-deficient cells. *N* = 4 (coverslips). White bars = 10 μm. Error bars are means ± SEM. *p < 0.5, **p < 0.01 using a two-way ANOVA and Sidak’s post test for **a**–**d** and a Student’s *t*-test for **e** and **f**

We next measured phagosomal proteolysis, using beads coated with Alexa-568 and DQ-OVA, a protease probe consisting of OVA molecules heavily labelled with self-quenching BODIPY dye, which becomes brightly fluorescent upon OVA hydrolysis^27^. DQ-OVA fluorescence increased progressively, and green fluorescence leaked from the phagosome into the cytosol, indicating that proteolysis occurred (Fig. 7a). Contrary to macrophages^27^, phagosomal proteolysis reported by this assay was only partially inhibited by ConcA (Fig. 7a). *Stim1* ablation significantly decreased DQ-OVA fluorescence by ∼30% at 90 min. This effect tended to persist in the presence of ConcA, although the difference here was not significant (*p* = 0.09) (Fig. 7a). Pre-loading cells with BAPTA-AM reduced proteolysis in both genotypes to the levels of untreated *Stim1* ^*−/−*^ cells, indicating that STIM1 mediates Ca^2+^ -dependent proteolysis in BMDCs (Fig. 7a). As an alternative approach, the levels of phagosome-associated OVA was determined by quantifying anti-OVA immunostaining of isolated BMDC phagosomes^28^. OVA degradation was inhibited in STIM1-deficient cells at 30 and 60 min but recovered after 2 h, indicating a partial or delayed OVA degradation defect (Fig. 7b, Supplementary Fig. 7a).

Since phagosome-to-cytosol transfer of peptides is suggested to be favoured by the delivery of ER proteins to phagosomes^16, 29^, we investigated whether endosome-to-cytosol transfer was influenced by *Stim1* ablation as a proxy measure of phagosomal antigen transfer^16^. BMDCs were loaded with the β-lactamase FRET reporter CCF4 and then exposed to β-lactamase. Upon transfer to the cytosol, β-lactamase cleaved CCF4, reducing the FRET emission in cells incubated at 37 °C with identical kinetics in WT and STIM1-deficient cells (Supplementary Fig. 7b, c). This suggests that defective phagosome-to-cytosol transfer likely cannot account for the decreased DQ-OVA signal and that delivery of the molecular machinery underlying phagosome-to-cytosol transfer may not be dependent on STIM1.

We then tested phago–lysosome fusion. BMDCs were pulsed with the FRET acceptor Alexa-568-HA for 3 h followed by an overnight chase to accumulate the dye in lysosomes^30^. OVAb coupled to the FRET donor Alexa-488 were then added for 30 and 90 min. The phagosome-associated FRET signal increased from 0 to 90 min in a ConcA and BAPTA-dependent manner, confirming that phago–lysosome fusion is Ca^2+^-dependent in BMDCs (Fig. 7c). Interestingly, *Stim1* ablation reduced phago–lysosome fusion by ∼30% at 90 min (Fig. 7c). Since OVA degradation was most strongly affected at earlier time points, we additionally tested whether endosome fusion might be defective, by pre-loading cells for 15 min with Alexa-594-dextran before exposing them to Alexa-488-OVAb and measuring the cellular FRET signal. Indeed, phago–endosome fusion was reduced in *Stim1* ^*−/−*^ cells at 15 and 30 min, with the effect most pronounced at 15 min (Fig. 7d). Addition of GSK (10 µM) together with OVAb abolished differences between WT and *Stim1* ^*−/−*^ cells (Fig. 7d). These data suggest that reduced phago–lysosome and phago–endosome fusion contribute to reduced proteolysis in STIM1-deficient cells. Therefore, we tested whether the proteolytic activity of lysosomal or endosomal proteases might be affected. While there was a trend for decreased activity of the lysosomal hydrolases involved in antigen processing^31^ cathepsin B/L (CatB/L), cathepsin S (CatS) and asparagine endopeptidase (AEP), the differences were not significant (Supplementary Fig. 7d). On the other hand, leucyl aminopeptidase activity, characteristic of the endosomal protease insulin-regulated aminopeptidase (IRAP) previously shown to be important for cross-presentation^32^, was reduced by ∼40% in phagosomes isolated from STIM1-deficient cells, but not in whole-cell lysates (Fig. 7e). Quantification of immunostainings revealed that periphagosomal IRAP was reduced by ∼20% (Fig. 7f). Together with the fact that IRAP^+^ endosomes are partially co-labelled with fluorescent dextran internalized for 15 min (Supplementary Fig. 7e), these data indicate that reduced fusion of IRAP-containing endosomes contributes to the cross-presentation defect observed in STIM1-deficient cells, although a direct effect on the activity of phagosomal IRAP or of other leucyl aminopeptidases cannot be excluded.

## Discussion

We report here that mice lacking STIM1 in the myeloid lineage fail to effectively cross-present phagocytosed antigens in vivo. The cross-presentation defect is recapitulated in vitro in DCs matured or not with TLR ligands, and is restricted to phagocytosed antigens, pointing to specific defects in phagocytic processing. Both global and localized Ca^2+^ signals are reduced in STIM1-deficient DCs. Surprisingly, but in line with a recent study^15^, DC differentiation, maturation and phagocytosis are not affected by *Stim1* ablation. Instead, defective cross-presentation is linked to two other impaired DC functions: defective in vivo migration, which correlates with reduced chemotaxis to Ca^2+^-mobilizing chemokines, and altered phagosomal maturation.

STIM1 deficiency reduces SOCE by 70% in both BMDCs and DC^2114^ cells indicating that STIM1 is the dominant isoform in DCs, and not STIM2 as proposed^13^. Since double *Stim1;Stim2* ablation completely abolishes SOCE in DCs^15^ however, STIM2 still contributes to Ca^2+^ signalling in DCs. Similar to Vaeth et al, and unlike human DCs^14^, we observe that *Stim1* is not required for the upregulation of DC maturation markers in mice, nor does it affect differentiation. Indeed, increased ROS production and upregulation of CCR7 and CXCR4, both events associated with DC maturation are additionally intact. This is in stark contrast to neutrophils derived from similar mouse genetic models, where phagocytosis^17, 25^ and ROS production^25^ are both STIM1 dependent. These differences highlight the importance of cell-type dependence of Ca^2+^ signalling outcomes. Moreover, in contrast to RAW macrophages^33^ and similar to B cells^34^, CpG does not induce detectable Ca^2+^ transients, while LPS induces small and variable Ca^2+^ transients in only ∼30% of cells. Thus, redundant Ca^2+^-dependent and independent pathways downstream of maturation stimuli appear sufficient to overcome a loss of STIM1 function in DCs.

We expected ROS production to be affected because NADPH oxidase activation is Ca^2+^ and STIM1 dependent in neutrophils^35^, and ROS production is required for effective cross-presentation (Fig. 1d and refs ^36–38^). Surprisingly ROS production is unaffected in STIM1-deficient DCs suggesting that either sufficient Ca^2+^ signalling remains, or that oxidase activation is SOCE-independent in DCs. Phagosomal pH is intimately linked to phagosomal ROS production and depends on the delivery of V-ATPase as well as HVCN1 proton channels^35, 39, 40^. In DCs phagosomal pH is controversial, with some groups reporting pH levels near 6.5 and others between 7 and 8 within the first 60 min of phagocytosis^36, 37^. In all previous reports phagosomal pH was measured on cell populations. Here pH measurements are conducted on single phagosomes, using two independent probes, FITC and pHrodo, comparing cells originating from the same mice. No differences in subpopulations or average pH are detected between STIM1 and wild-type cells, but the two dyes yield an average pH differing by nearly 1.0 unit. This likely reflects the lower in situ pKa of pHrodo (6.1 ± 0.4 vs. 7.6 ± 0.3 for FITC) and the increased sensitivity of pHrodo at low pH levels (Supplementary Fig. 6b), which indicates that pHrodo reports a more reliable pH estimate. On the other hand, the differences could also be related to the large variability in phagosomal pH that appears to be regulated at the level of single phagosomes. Shifts in the balance between the peaks of this bimodal, non-Gaussian distribution between experimental conditions may explain why different groups report such disparate values for mean population measurements. Why DC phagosomes display such a large variability remains unclear but phagosomal ROS, which can be highly heterogenous^41^, and can inhibit V-ATPase recruitment^35^, is likely involved. Indeed, *Hvcn1* ^*−/−*^ neutrophils, which suffer a 50% loss in ROS production, also display a bimodal distribution of phagosomal pH^39^, and thus it could be interesting to correlate ROS and pH measurements at the level of single phagosomes in future studies in DCs. It is interesting to note that ORAI channels are sensitive to extracellular acidity, being activated at alkaline pH while inhibited at pH < 6^42^. The heterogeneity of phagosomal pH observed would thus allow phagosomes with robust ORAI activity to co-exist with phagosomes where ORAI is inhibited, and could explain why only a subset of STIM1-positive periphagosomal puncta coincide with Ca^2+^ hotspots. The identical pH profiles of STIM1 and wild-type cells however, indicate that altered pH cannot account for defects in cross-presentation.

Despite the lack of differences in either ROS or pH, STIM1-deficient DCs display surprisingly lower levels of proteolysis, measured by two independent assays, as well as reduced leucyl aminopeptidase activity, characteristic of the endosomal protease IRAP previously implicated in cross-presentation^32^. Decreased proteolysis is associated with decreased phago–endolysosome fusion, decreased global and periphagosomal Ca^2+^ signals, and decreased frequency of ER–Ph MCS in STIM1-deficient cells. Although we cannot differentiate whether loss of global or local Ca2^+^ signals cause reduced fusion events, the high vesicular fusion/fission activity near ER–Ph MCS favours a model where local Ca^2+^ signals promote local fusion events. It should be noted that decreased cathepsin-dependent proteolysis and reduced phago–lysosome fusion correlated with higher, not lower, levels of cross-presentation in DCs^38, 43^. Yet, some proteolysis must occur to generate peptides from whole OVA molecules and inhibiting proteolysis with the broad-spectrum inhibitor leupeptin reduces cross-presentation in some^44–46^ although not all^47, 48^ contexts. Indeed, in the companion study published in this same issue which analyses the function of UNC93B1, a novel STIM1 interactor^49^, Maschalidi et al. not only confirm a role for STIM1 in cross-presentation, but also observe that decreased proteolysis correlates with decreased cross-presentation. Proteolysis has therefore a bipartite effect on antigen processing, with both negative and positive effects on the MHC-I loading depending on its extent. Our data suggest that finely regulated levels of proteolysis must be reached to favour cross-presentation, and that this fine-tuning is achieved at least in part through Ca^2+^-dependent regulation of endosome fusion and IRAP delivery.

We also considered that antigen export from phagosomes into the cytosol might be affected by STIM1 deletion. Phagosomal export was reported for some but not all antigens^50^ and cytosol transfer requires ER proteins such as Sec61, tapasin and TAP^1^. That STIM1 mediates close contacts between the ER and phagosomes argues that at least some ER proteins found on isolated phagosomes are derived from contacts, which may in turn facilitate the delivery of this ER-derived machinery. That the frequency of ER–Ph MCS was fivefold higher in WT DCs as compared to WT neutrophils, yet the frequency of periphagosomal Ca^2+^ signals is only half that of neutrophils^17^, as well as the observation of periphagosomal STIM1 puncta without associated Ca^2+^ signals (Fig. 3e), further support the idea that ER–Ph MCS may have roles beyond Ca^2+^ signalling in DCs, and that they could partly mediate specialized DC functions that rely on ER proteins. However, no defects in cytosol transfer were observed. On one hand, ER contacts were only decreased by ∼35% upon *Stim1* deletion, and potentially, sufficient activity remained to allow effective cytosol transfer. On the other hand, recent data suggests that different ER proteins such as tapasin^16^, MHC-I^8^ and Sec61^7^ reach phagosomes through unconventional vesicular trafficking pathways involving the ERGIC, recycling endosome and possibly other compartments. These new findings on alternative trafficking raise the intriguing possibility that endomembrane trafficking is more generally non-canonical in DCs and that specialized compartments analogous to secondary and tertiary granules in neutrophils might exists in DCs. Indeed, a lysosome-like Rab34-dependent compartment delivers NADPH oxidase in DCs^48^. Since cross-presentation was more strongly impaired than phago–lysosome fusion, STIM1 might regulate alternative Ca^2+^-dependent trafficking pathways in a way that bypasses the need for cytosol transfer. The generation of better tools to manipulate these pathways will be required to gain a deeper understanding of alternative compartments and the signals that regulate their activity.

In summary, we show that STIM1 has an important and selective regulatory role on Ca^2+^-dependent processes that control phagosome maturation and migration in DCs, thereby impacting the efficiency of cross-presentation. STIM-gated ORAI channels are promising drug targets for treating autoimmune diseases^51^, muscle defects^52^, skin disorders^53, 54^ and cancer^55^. In addition, activating ORAI1 through a genetically encoded light-inducible STIM1 fragment promotes tumour remission in a mouse model of DC cell-based immunotherapy^56^. Together with our data showing that either pharmacological or genetic manipulation of Ca^2+^ signalling can decrease cross-presentation, this suggests that delineating the precise role of SOCE in innate immune cells is highly relevant for the identification of potential risks and benefits associated with the development of SOCE blockers and activators designed for therapeutic use.

## Methods

### Reagents

The following antibodies (antibody Name/catalogue#/dilution) were purchased from: Biolegend (USA)- PerCP/Cy5.5-anti-CD197 (also known as CCR7)/120115/1:100; PE-anti-CD184 (also known as CXCR4) L276F12/146505/1:100; APC-anti-F4/80 (BM8)/123115/1:400, APC-Rat-IgG2a, κ Isotype Ctrl/400511/1:100; PE/Cy7-anti-CD11b (M1/70)/101225/1:200; PE-anti-CD86 (GL-1)/105007/1:200; PE/Cy7-anti-CD40 (3/23)/124621/1:100; APC-anti-CD80 (16-10A1)/104713/1:100; Alexa-647-anti-I-Ab (also known as MHC-II, KH74)/115309/1:200; PE/Cy7-anti-CD8α (53-6.7)/100721/1:200; APC-anti-CD45.1 (also known as PTPRC^a^, A20)/110713/1:100. BD Biosciences (USA): APC-anti-CD11c (HL3)/561119/1:100; anti-CD16/CD32 (2.4G2) “FcBlock”/553142/1:200; anti-STIM1/610954/1:100. Millipore (USA): anti-STIM1/AB9870/1:1000. eBiosciences (USA): PE-anti-CD11c (N418)/17-0114-81/1:100; APC-anti-CD45.2(104)/17-0454-81/1:100. Cell Signalling (USA): anti-STIM2/4917S/1:1000; anti-IRAP/6918/1:250. Sigma-Aldrich (Germany): anti-Orai1/O8264/1:500. Bio-Rad (USA): anti-mouse-HRP/1706516/1:10,000; anti-rabbit-HRP/ 172101/1:10,000. Jackson ImmunoResearch (USA): Alexa-488-anti-mouse /715-545-150/1:800. Recombinant murine SDF-1α (also known as CXCL12), recombinant murine CCL21 (also known as Exodus-2 or SLC), and recombinant murine GM-CSF were obtained from Peprotech (UK). Carboxyfluorescein succinimidyl ester (CFSE) was obtained from eBiosciences. Mouse shSTIM1 (TRC Clone ID: NM_009287.2-2712s1c1 Sequence: CCGGCCCTTCCTTTCTTTGCAATATCTCGAGATATTGCAAAGAAAGGAAGGGTTTTT TG) and shCTR (Non-target Control particles SCH002V) Mission shRNA Lentiviral Clones were purchased from Sigma-Aldrich. All lentiviral particles were produced in Lenti-X 293T cells using the Lenti-X HTS Packaging System (Takara, Japan) according to the manufacturer’s instructions. Lentiviral titres were determined using the LentiX-p24 Rapid Titre ELISA kit (Takara). All cell culture reagents were obtained from ThermoFisher Scientific, and all chemicals were purchased from Sigma-Aldrich unless otherwise stated. mCherry-STIM1^57^ cloned into pENTR1a was purchased from GenScript (USA). mCherry-STIM1 was then cloned into the p2K7_bsd_ lentiviral vector containing the ubiquitin promoter using the Gateway LR clonase (ThermoFisher Scientific, USA) following the protocol described in ref. ^58^. OVA-beads were prepared by washing 3.0 μm unlabelled or YG-Fluoresbrite polystyrene microspheres (Polysciences, USA) in 25 mM sodium citrate, 25 mM sodium phosphate buffer, pH 5.0, and incubating beads with 20 mg mL^−1^ endotoxin-free chicken ovalbumin (OVA, InvivoGen, USA) overnight at 4 °C on an end-over-end rotor, followed by three washes in sterile PBS (ThermoFisher).

### Flow cytometry

Cells were washed once in ice cold FACS buffer (2% BSA, 20 mM EDTA in PBS), blocked with 1:200 FcBlock/FACS buffer 15 min on ice, incubated for 30 min–1 h with the indicated antibodies, and washed with FACS buffer. Fluorescence was analysed using an Accuri C6 flow cytometer and CFlow Plus software (BD Biosciences) unless otherwise indicated.

### Mice, cells and transduction

Mice on a C57BL/6 background bearing a conditional knockout of the *Stim1* gene in the myeloid lineage were generated from LysM-Cre (B6.129P2-*Lyz2* ^*tm1(cre)Ifo*^/J^59^, The Jackson Laboratory, USA) and *Stim1* ^*fl/fl*^ (B6(Cg)-*Stim1* ^*tm1Rao*^/J^60^, (a kind gift from Dr. Masatsugu Oh-Hora) strains, and genotyped from ear biopsies using a KAPA Mouse Genotyping Kit (Sigma) according to the manufacturer’s instructions, and the following primers and PCR conditions: For *Stim1*, CGATGGTCTCACGGTCTCTAGTTTC; AACGTCTTGCAGTTGCTGTAGGC; GGCTCTGCTGACCTGGAACTATAGTG and 94 °C 3 min, 94 °C 7 s, 60 °C 20 s, 72 °C 25 s, 30 cycles, 72 °C 3 min. For LysM-Cre: CCCAGAAATGCCAGATTACG, CTTGGGCTGCCAGAATTTCTC, TTACAGTCGGCCAGGCTGAC and 95 °C 5 min, 95 °C 45 s, 60 °C 20 s, 72 °C 25 s, 40 cycles, 72 °C 5 min in separate reactions, where 1% DMSO and 1.6% Perfect Match (Agilent) were added to the reaction of the mutant band. Bone marrow was isolated from sex and age-matched 6–16 week-old males and females. All animal manipulations were approved by the Geneva canton’s Direction Générale de la Santé (authorizations GE/87/15 and GE/142/16) and performed in accordance to the guidelines of the animal research committee at the University of Geneva. BMDCs were generated by culturing bone marrow cells isolated from mouse femurs and tibias, where red blood cells had been removed by lysis in ammonium buffer (155 mM NH_4_Cl, 10 mM KHCO_3_, 0.1 mM EDTA), in DMEM (41965-039) supplemented with 10% endotoxin-free FCS, 50 μM β-mercaptoethanol, 1% sodium pyruvate, 1% penicillin/streptomycin and 20 ng mL^−1^ murine recombinant GM-CSF. Cells were used between 8 and 15 days of culture. The purity of each culture was determined to be > 85% CD11c^+^ by flow cytometry. The DC^2114^ cell line, derived from C57BL/6 mice with an H2-K^b^ haplotype^18^ was cultured in IMDM (31980) supplemented with 10% FCS, 50 μM β-mercaptoethanol, and 1% penicillin/streptomycin. Cells were tested for mycoplasma every six months. Maturation was performed by incubating cells in either 1 μg mL^−1^ LPS or 0.1 μM CpG (ODN 1826, InvivoGen) for 18 h. Seeding cells on coverslips tended to activate them to variable extents, thus unless otherwise indicated cells seeded on coverslips were matured with CpG. Lentivirus transduction was performed by centrifuging cells and viral particles at 5 MOI in complete medium supplemented with 8 μg mL^−1^ polybrene at 500 g at 37 °C for 1 h. To produce DC^2114^ cells stably expressing shRNA, cells were cultured in 1 μg mL^−1^ puromycin as of 2 days after transduction. Splenic OT-I cells were isolated from C57BL/6-Tg(TcraTcrb)1100Mjb/J mice^61^ (Jackson) using the CD8α^+^ negative selection mouse T cell isolation kit (Miltenyi Biotec, Germany) according to the manufacturer’s instructions. Congenic CD45.1^+^ OT-I cells were obtained by breeding OT-I mice to the congenic CD45.1 strain B6.SJL-Ptprc^a^ Pepc^b^/BoyJ^62^ (Jackson) and similarly isolating CD8α^+^ cells. Cell purity was verified to be >95% CD8α^+^, CD4^−^ by flow cytometry.

### In vivo cross-presentation

Three to four-month-old female *LysM-Cre* ^*ki/+*^ *;Stim1* ^*fl/fl*^ and otherwise wild-type *Stim1* ^*fl/fl*^ littermates were anesthetized with isofluorane and injected with 50 μl of 1.0% OVA-beads/PBS on the left and 0.5% OVA-beads/PBS on the right footpads, or with only PBS on both footpads as control. Twenty four hours later the mice were retro-orbitally injected with 1 × 10^6^ CD45.1^+^ OT-I cells labelled with 3.5 μM CFSE for 10 min and washed and resuspended in 50 μl sterile PBS. After 72 h, the mice were sacrificed and popliteal draining and axial/brachial non-draining lymph nodes were harvested in FACS buffer containing 2% FCS. Lymph nodes were mechanically crushed onto 40 μm cell strainers and the cellular filtrate blocked and stained as described above with anti-CD8α-PE/Cy7 anti-CD45.1-APC antibodies. The total number of CD45.1^+^ cells as well as their CFSE fluorescence was determined for 50,000 CD8α^+^ cells. %Proliferation is defined at the percentage of cells showing diluted levels of CFSE fluorescence as compared to uninjected controls.

### In vitro cross-presentation

BMDCs were seeded at 20,000 cells per well and DC^2114^ at 10,000 cells per well in black/clear-bottom 96-well plates (Greiner, for BrDU-based assays) or in round-bottom 96-well plates (Corning, for CFSE assays), and allowed to adhere for 24 h. For 3:1 and 10:1 T cell:DC ratios, BMDCs were diluted accordingly. Cells were exposed to either OVAb at 20:1 ratio for 4 h or SIINFEKL peptide (InvivoGen) for 1 h at the given concentrations, before wells were washed in complete medium, and irradiated with 3000 cGy using a Gammacell 3000 Elan Ce^127^ irradiator (Best Theratronics, Canada). 20,000 (for BMDC) or 10,000 (for DC^2114^) OT-I cells were added to each well, and plates were incubated at 37 °C/5%CO_2_ for 72 h. T cell proliferation was assessed by BrDU incorporation using a Cell Proliferation ELISA, BrDU (Chemiluminescent) kit (Roche, Switzerland) according to the manufacturer’s instructions and using a SpectraMAX Paradigm (Molecular Devices, USA) plate reader. Proliferation of OT-I cells labelled with CFSE was assessed as described above.

### In vivo migration

BMDCs isolated from *LysM-Stim1* ^*fl/fl*^ and from Ubiquitin-eGFP mice (C57BL/6-Tg(UBC-GFP)30Scha/J^63^, Jackson) were washed and resuspended in PBS containing 0.5% OVAb at 20 × 10^6^ cells per mL each. In total, 50 μl of cell/bead mixture were injected into left and right footpads of congenic B6 CD45.1^+^ mice. Popliteal lymph nodes were harvested 24 h or 48 h post injection and DCs were recovered from LN after digestion in an enzymatic mix containing collagenase D (1 mg mL^−1^) and DNAse I (10 μg mL^−1^) (Roche) in HBSS. Total LN cells were incubated with FcBlock for 10 min at 4 °C and stained with PE-Cy7-anti-CD11c, APC-anti-CD45.2 and Brilliant-Violet-anti-CD45.1. Migrating CD11c^high^ CD45.2^+^ DCs were quantified by flow cytometry with a CyanTM ADP (Beckman Coulter) and analysed using FlowJo software (FlowJo Company, USA).

### In vitro transwell migration assay

BMDCs were washed and resuspended in migration medium (1% BSA, 10% serum in phenol-free DMEM). 5 × 10^5^ cells were seeded onto 8.0 μm pore cell culture inserts in a 24-well plate (Corning), and either control medium or medium supplemented with 10^−6^ M chemoattractant (fMIFL, SDF-1α or CCL21) was added to the bottom chamber to initiate migration. Cells were incubated at 37 °C for 24 h, after which inserts were removed and plates were placed on ice, 5 mM EDTA was added to each well, and nuclei were stained with 2.5 μM DRAQ5 (ThermoFisher). The total number of migrated DRAQ5^+^ cells was quantified. % Migration is defined as the total number of migrated cells in response to chemoattractant divided by the total number of migrated cells in response to control medium minus 1 times 100%.

### Phagocytosis

For flow cytometry-based phagocytosis assays, OVA-coated YG-Fluoresbrite beads were added at a 20:1 ratio in duplicate for the indicated times. Cells were washed in FACS buffer and stained with anti-CD11c-APC antibodies as described above, and the mean fluorescence intensity (MFI) quantified for CD11c^+^ cells. MFIs were normalized to the mean WT 4 h phagocytosis value performed on the same day. For microscopy-based phagocytosis assays cells were matured with CpG, exposed to 20:1 OVAb for 4 h, washed, fixed in 4% paraformaldehyde/PBS, permeabilized and blocked in 0.1% triton-X/1% BSA, stained with 0.6 μM TRITC-phalloidin and 10 μg mL^−1^ Hoechst 33342 and visualized using confocal microscopy.

### Immunofluorescence

Immunostaining of BMDCs with anti-STIM1 was performed as described^17^ except that cells were fixed for 10 min in 2% paraformaldehyde(PFA)/PBS. Briefly, cells were permeabilized in 0.1% Triton-X for 5 min, washed 3× PBS, reduced with 0.1% NaBH_4_/PBS, blocked with Image-IT-FX (Thermo) for 20 min, blocked with 0.5% BSA/PBS+FcBlock for 30 min, incubated overnight in primary antibody and for 1 h in secondary antibody in 0.5% BSA/PBS. For anti-IRAP immunostainings, cells were exposed to either 20:1 Alexa-488 OVAb or lysine-fixable FITC-dextran (10,000 kDa, 50 µg mL^−1^) for 15 min prior to fixation for 10 or 30 min, respectively, in 4% (PFA/PBS). For OVAb stainings, cells were then blocked in 0.5%BSA/0.2% saponin/PBS+FcBlock for 30 min and primary and secondary antibodies applied as above in the same buffer. For dextran stainings, cells were permeabilized in 0.01% Triton-X/PBS for 3 min, washed 3× in PBS, blocked in 0.5% BSA/PBS+FcBlock, and similarly incubated with primary and secondary antibodies.

### Ca^2+^ imaging

BMDCs and DC^2114^ cells seeded on Fluorodishes (WPI, USA) and, where indicated, matured as described above. Cells were loaded with 4 μM Fura-2-AM, 0.01% pluronic (ThermoFisher) in modified Ringer’s^17^ for 30 min at room temperature (RT). 340/380 nm excitation 510± 40 nm emission ratiometric imaging was performed at 37 °C in modified Ringer’s where Ca^2+^-free solution contained 1 mM EGTA instead of 2 mM CaCl_2_. Frames were acquired every 3 s. Ca^2+^ microdomain/hotspot imaging was performed as described^17^ except cells were loaded with 4 μM Fluo-8-AM (AAT Bioquest) for 30 min at 37 °C, 30 min at RT and 2.5 μM BAPTA-AM for the last 10 min, in modified Ringer’s containing 500 μM sulfinpyrazone. Simultaneous excitation at 488 and 543 nm and emission collection in two separate channels for green (Fluo8) and red (mCherry) was used. For quantification of periphagosomal Ca^2+^ hotspots, images were averaged over 6 s and captured between 20 and 30 min after the addition of OVAb. Where applicable Xestospongin C (1 μM) was applied 20 min after exposure of BMDCs to OVAb and hotspots imaged at 30 min, while GSK7975A (a gift from Dr. Martin Lochner, University of Bern, 10 μM) was applied concurrently with OVAb and images taken after 30 min.

### Western blotting

Cells were washed with ice cold PBS and lysates prepared as described^17^. Total protein was quantified using a BCA assay (ThermoFisher) according to the manufacturer’s instructions. 50 μg per lane were loaded for ORAI1 gels, while 30 μg per lane were loaded for all others. 4–20% Mini-Protean TGX Pre-cast gels (BioRad) and iBlot PVDF kits (ThermoFisher) were used for SDS-PAGE and transfer respectively. All antibodies were diluted in 3% milk/1%Tween/TBS, primary antibodies were incubated overnight and secondary antibodies incubated for 1 h. Membranes were visualized using Immobilon Western Chemiluminescent HRP Substrate (Millipore) and a PXi gel imaging system (Syngene, UK).

### Electron microscopy

Classic transmission electron microscopy (TEM) and 3D Focused-ion beam scanning electron microscopy (FIB-SEM) were performed with the help of the Electron Microscopy Core Facility of the University of Geneva. TEM samples were prepared and imaged as described^17^. FIB-SEM was performed on a Helios NanoLab G3 microscope (FEI, Netherlands) with images acquired using the electron beam at 2 kV/0.2 nA, and milling sections with the ion beam at 30 kV/2.5 nA every 10 nm. Specimens were prepared using a protocol modified from^64^. Cells were fixed in 2.5% glutaraldehyde, 2% PFA in 2 mM CaCl_2_ in 0.15 M cacodylate buffer (pH 7.4) (Ca-Caco) for 3 h on ice and washed 5× in cold Ca-Caco. Samples were incubated for 1 h on ice in 2% OsO_4_, 1.5% KFCN in Ca-Caco and washed 5× in cold ddH_2_O. Samples were then incubated in a freshly prepared and filtered 1% thiocarbohydrazide for 20 min at RT, washed 5× in RT ddH_2_O, incubated in 2% OsO_4_ for 30 min at RT and then incubated in cold 1% uranyl acetate at 4 °C overnight. Samples were then washed 5× in RT ddH_2_O and stained using Walton’s lead aspartate method for 30 min at 60 °C, and rinsed 5× in RT ddH_2_O. Samples were dehydrated in 2 × 7 min steps of cold 20, 50, 70, 90, 100 and 100% anhydrous ethanol, then cold anhydrous acetone for 10 min and RT acetone for 10 min. Samples were then embedded in hard epon resin by infiltrating with 25, 50 and 75% hard epon/acetone at RT for 2 h each, then 100% hard epon overnight. Fresh 100% hard epon was exchanged the next day incubated for 3 h. Epon was once more exchanged before polymerization for 48 h at 60 °C. Cut and microtome-trimmed blocks were mounted on aluminium pins using Pelco C100 cyanoacrylate glue and Pelco conductive silver paint (Ted Pella, USA). Samples were sputter-coated with gold for 20 s using a Q150T ES coater (Quorum Technologies, UK).

### pH measurements

Phagosomal pH was measured using FITC-coupled zymosan added at 20:1 ratio as described^65^ except that calibrations (pH 4.0–9.0) were performed using five different stacks per condition for each calibration buffer rather than a single time-lapse acquisition. pHrodo Red Zymosan Bioparticles (ThermoFisher) were washed 3× in sterile filtered 0.1 M NaCO_3_ buffer (pH 8.0), 10 μg mL^−1^ Alexa-488-succinimidyl ester (SE) was added and particles were agitated for 4 h at RT. Unbound dye was removed by washing in 50, 33%, then 25% DMSO/PBS and 3× in sterile PBS. Zymosan was then incubated in 20 mg mL^−1^ OVA overnight and washed 3× in PBS. Measurements, calibration and analysis were obtained using an identical protocol to FITC-coupled zymosan with the following exceptions: imaging was performed using alternate 555/590 and 488/530 nm Ex/Em illumination. Zymosan were added to cells in complete medium and were washed in modified Ringer’s after 30 min, and imaged 30 and/or 90 min after addition. Where applicable, 0.2 nM ConcA was added immediately before zymosan. Endosomal pH measurements were made as previously described^66^. Briefly, cells were loaded with with 1 mg mL^−1^ of FITC- and Alexa-647-labelled 40 kDa dextrans (Thermo) for 10 min at 37 °C and extensively washed with cold PBS/1% BSA. Cells were then chased for different times and analysed by flow cytometry.

### ROS measurements

Extracellular ROS were measured on cells seeded at 50,000 cells per well in black/clear bottom plates using the Amplex Red Ultra Hydrogen Peroxide/Peroxidase kit (ThermoFisher), according to the manufacturer’s instructions. Intracellular ROS were measured on cells similarly plated. Dihydroethidium (DHE) (30 μM) was loaded for 1 h in complete medium at 37 °C before washing in modified Ringer’s. For both assays fluorescence was measured every 2 min for 90 min at 37 °C after addition of the indicated stimuli (100 nM PMA, 20:1 zymosan or 20:1 OVAb) using a SpectraMAX Paradigm plate reader, and is reported as the maximum fluorescence value normalized to the average baseline value over the first 6 min. Intraphagosomal/intracellular ROS was measured on cells plated on Fluorodishes using OxyBurst/Alexa-568-coupled beads. 3.0 μm Polybead Amino Microspheres (Polysciences) were washed in 3× in sterile filtered 0.1 M NaCO_3_ buffer (pH 8.0), 20 μg mL^−1^ OxyBurst-H2DCFDA-SE (ThermoFisher) was added and particles were agitated for overnight at RT in tubes filled with nitrogen. Alexa-568-SE (ThermoFisher) was then added for 1 h. Unbound dye was removed as above; beads were coated with OVA as above and beads were stored under nitrogen. OxyBurst/Alexa568-OVA beads were added to cells at 20:1 in complete medium, were washed in modified Ringer’s after 30 min, and imaged using alternate 555/590, 488/530 nm Ex/Em and brightfield illumination 30 and/or 90 min after addition. Where applicable cells were pre-incubated for 10 min with 10 μM DPI prior to stimulus addition. As OxyBurst leaked from phagosomes into the cytosol, brightfield images were used to demarcate cell borders and the ratio of whole-cell OxyBurst to whole-cell Alexa-568 fluorescence minus the basal (external bead) ratio calculated as an index of intracellular/phagosomal ROS.

### Measurement of cytosol transfer

Measurement of cytosol transfer was performed as previously described^16^. Briefly, cells were loaded with 4 μM CCF4-AM probe (a FRET-sensitive cytosolic substrate of β-lactamase, ThermoFisher) for 1 h, washed with PBS and incubated at 37 °C with 2 mg mL^−1^ β-lactamase for different time points. Reactions were stopped with cold PBS and live, single, CD11c^+^ cells were analysed on a BD Biosciences Fortessa flow cytometer by monitoring the increase in 450 nm fluorescence and loss of 535 nm emission fluorescence resulting from CCF4 cleavage.

### Proteolysis assays

Phagosomal proteolysis was measured using a protocol modified from ref. ^30^. 3.0 μm Polybead Amino Microspheres (Polysciences) were labelled with Alexa-568-SE as described above. Labelled beads were then washed in 25 mM sodium citrate, 25 mM sodium phosphate buffer (pH 5.0), and incubated with 20 mg mL^−1^ DQ-ovalbumin (DQ-OVA, ThermoFisher) overnight at 4 °C on an end-over-end rotor, followed by three washes in sterile PBS (ThermoFisher). Cells were exposed to beads, imaged and analysed as described above for OxyBurst assays. Where applicable cells were pre-loaded for 30 min with 40 μM BAPTA-AM, and washed prior to stimulus addition, and 0.2 nM ConcA added just prior to beads.

### Phagosomal OVA degradation assay

Phagosomal OVA degradation assay was performed as previously described^66^. Briefly, 3.0 µm Polybead Amino Microspheres (Polysciences) were covalently coated with OVA (0.2 mg mL^−1^) according to the manufacturer’s instructions. BMDCs were pulsed for 15 min at 37 °C and chased for different times at 37 °C. Cells were lysed in 50 mM Tris-HCl (pH 7.4) supplemented with 150 mM NaCl, 1 mM DTT, 0.5% NP-40, and cocktail of protease inhibitors (Roche). For flow cytometry experiments, beads were stained with polyclonal anti-OVA (Sigma) and a secondary anti-rabbit Alexa488 (Thermo), and analysed on a BD Biosciences Fortessa flow cytometer.

### Protease activity assay

Cathepsin/AEP protease activity assays were performed as previously described^66^ using 1 µg of protein in lysis buffer (50 mM Tris/150 mM NaCl/1% NP40) on a Mithras LB 940 microplate reader by measuring the release of fluorescent N-Acetyl-Methyl-Coumarin substrate (100 µM) in citrate buffer (pH 5.5) at 37 °C, detected using 380/440 nm excitation/emission. Leucyl aminopeptidase activity was measured as previously described^67^ in 50 mM Tris buffer, pH 7.5/1 mM DTT and 100 µM substrate except that activity was measured on 3 µg of whole-cell or purified phagosome lysates without anti-IRAP immunoprecipitation. Specific substrates for AEP (Z-Ala-Ala-Asn- NHMec), CatB/L (Z-Phe-Arg-NHMec), CatS (Z-Val-Val-Arg-NHMec) and IRAP substrate (Leu-AMC) were purchased from Bachem.

### Phagosome isolation for leucyl aminopeptidase activity

1.5 × 10^7^ BMDCs were exposed to 3 × 10^8^ magnetic 3 µm OVA-coated beads (Polysciences) for 15 min on ice. Warm medium was added and cells were incubated for 20 min at 37 °C. Cells were returned to ice and washed twice in cold PBS and once in cold IEB buffer containing protease inhibitor cocktail (PI, Sigma) (IEB: 10 mM HEPES, 1 mM EGTA, 25 mM KCL, 250 mM sucrose). Cells were scraped, centrifuged, and washed once in IEB/PI and counted. Cells were then centrifuged and resuspended in IEB/PI at a concentration of 4 × 10^6^ cells mL^−1^, and passed 20x through a 20 µm clearance ball-bearing homogenizer. Homogenates were then transferred to a magnet and washed once in IEB/PI, once in IEB/PI + 4 mM MgCl_2_ + 10 mM Na-ATP, and were then incubated on an end-over-end rotor for 15 min at 4 °C. Homogenates were washed one final time on a magnet and resuspended in lysis buffer (50 mM Tris; 150 mM NaCl; 1% NP40) without protease inhibitors. Lysates were incubated on ice for 30 min prior to quantification using a BCA kit (ThermoFisher).

### Phago–lysosome and phago–endosome fusion assay

Phago–lysosome fusion was measured using a protocol modified from^30^. 3.0 μm Polybead Amino Microspheres (Polysciences) were labelled with Alexa-488-SE and coated with OVA as described above. Twenty four hours prior to the assay cells were pulse labelled with 20 μg mL^−1^ Alexa-594-HA (ThermoFisher) in complete medium for 3 h, washed, and followed by a chase period of 24 h to allow the dye to accumulate in lysosomes. Alexa488-OVA beads were added to cells at 20:1 in complete medium, were washed in modified Ringer’s after 30 min, and imaged using alternate 555/590, 488/530, 488/590 nm Ex/Em and brightfield illumination 30 and/or 90 min after addition. When applicable cells were pre-loaded for 30 min with 40 μM BAPTA-AM, and washed prior to stimulus addition and 0.2 nM ConcA added just prior to beads. The P–L fusion index is computed as the ratio of the FRET (488/590)/Alexa-488 (488/530) signal minus the basal (external bead) FRET signal, normalized to the total average cellular Alexa-594-HA fluorescence. Phago–endosome fusion assays were performed in a similar manner except that 1 μg mL^−1^ Alexa-594-dextran of 10,000 MW (ThermoFisher) was added 15 min prior to beads, cells were washed 3× in complete medium prior to Alexa488-OVA bead addition and external beads were washed in modified Ringer’s after 15 min. Where applicable, GSK7975A (10 μM) was added concurrently with beads.

### Image analysis and statistics

All fluorescence image analyses were performed using ImageJ software (NIH) on maximum projections of at least 5 × 15 μm *z*-stacks per condition, except for IRAP quantification which were performed on a single confocal plane from each stack, and which were taken with a 100× objective. Semi-automated segmentation based on a single visually determined threshold for the pH/ROS/proteolysis insensitive wavelength did not fully separate all closely clustered-phagosomes particles, and thus single-phagosome measurements include an estimated 20–30% phagosome clusters. External (non-cell-associated) zymosan/beads were used to estimate time 0 ratio values and then eliminated manually from the analysis. For IRAP quantification, the total periphagosomal IRAP signal (obtained from the bead segmentation mask, and measured in a single plane that captured most phagosomes within a stack at their mid-section) was normalized to the total IRAP cellular fluorescence, calculated from sum projections of the same stack. Alignment, segmentation and 3D reconstruction of FIB-SEM images was performed using Amira software (FEI). Quantification of ER–Ph MCS was conducted in single-blind and performed manually on 10 nm slices, at 30 slice intervals to avoid double counting of MCS. MCS were defined as areas where the ER was < 30 nm away from the phagosomal membrane. Phagosome cross-sections smaller than 1.5 µm in diameter were excluded from the analysis to avoid any under-sampling bias of phagosomal membranes. MCS frequency was defined as the number of MCS (also called ER junctions) detected divided by the total number of phagosomes analysed, multiplied by 100 to obtain the average number of ER junctions per 100 phagosomes. Animal studies were not conducted blind, but at least *N* = 4 animals were tested for each condition, and, within each genotype, were randomly assigned to control and experimental groups. All statistical analyses were performed using Prism 6.0 software (GraphPad). Pairwise comparisons were made using a Mann-Whitney test for experiments based on phagocytosis, as phagocytic events do not follow a Gaussian distribution. A two-sided Student’s *t*-test was used for all other pairwise comparisons, and, where *F*-test showed significantly difference variances, Welch’s correction was applied. A Two-way ANOVA with Sidak’s multiple comparisons test was used for repeated measures. *N* = number of independent experiments, at least three independent experiments were performed for all conditions.

### Data availability

The data that support the findings of this study are available from the corresponding author upon reasonable request.

## Electronic supplementary material

Supplementary Information

Peer Review File

Description of Additional Supplementary Files

Supplementary Movie 1
