# Supplementary material for: STIM1 promotes migration, phagosomal maturation and antigen cross-presentation in dendritic cells
Source: Nat Commun. 2017 Nov 24;8:1852. doi: 10.1038/s41467-017-01600-6 (PMC5701258; doi:10.1038/s41467-017-01600-6)
Supplement: Supplementary file 1 — Supplementary Information [file 41467_2017_1600_MOESM1_ESM.pdf]

Supplementary Fig. 1

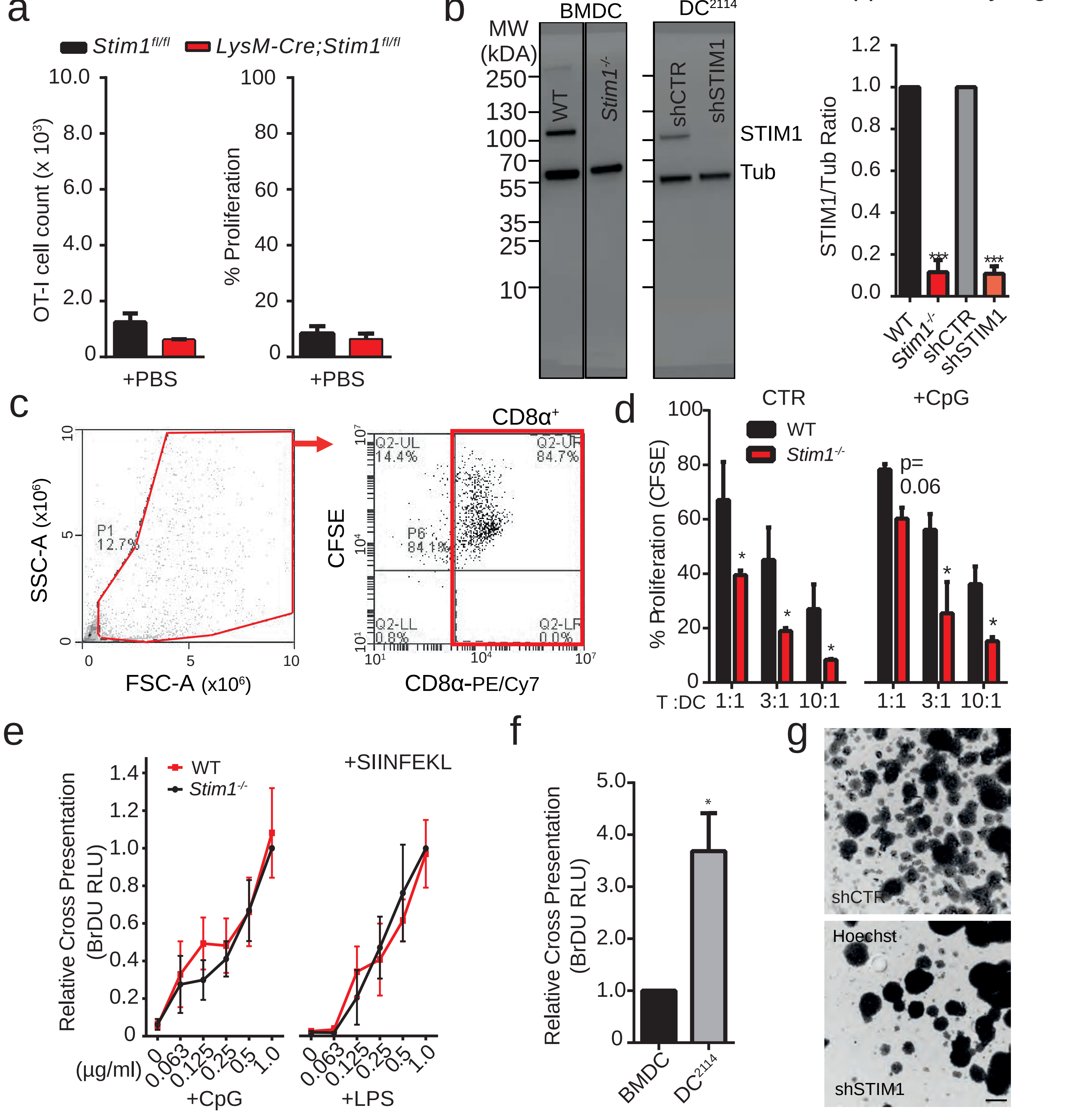

Supplementary Fig. 1. a) Isolating draining lymph nodes of *LysM-Cre;Stim1*$^{fl/fl}$ mice or wild-type *Stim1*$^{fl/fl}$ littermates injected with PBS only, and with 1x10^{6} CD45.1^{+} CFSE-labelled OT-I cells revealed no differences in the total number of CD45.1^{+} OT-I cells (left) nor in their proliferation (right). The gating strategy is shown in Fig. 1a. See also Fig. 1b. **b)** Western blots show a potent reduction in STIM1 expression both in *Stim1*$^{-/-}$ BMDCs as compared to WT, and in DC^{2114} cells stably transduced with shSTIM1 as compared to shCTR. N=3. Values are STIM1 to Tubulin (Tub) ratios normalized to WT or shCTR. **c)** Full gating strategy for panel d. **d)** Co-culture of BMDCs and CFSE-labelled OT-I cells at varying ratios confirmed that STIM1 ablation reduces cross-presentation both in immature (CTR) and mature (CpG) cells. N=3, triplicate wells. **e)** Cross-presentation, assessed by BrDU, of WT BMDCs matured or not with CpG or LPS, exposed to varying concentration of SIINFEKL peptide and co-cultured 1:1 with OT-I cells. No significant differences between WT and *Stim1*$^{-/-}$ BMDCs were detected. N = 3, triplicate wells. **f)** Cross-presentation, assessed by BrDU, of WT BMDCs (20,000 cells/well) or DC^{2114}+shCTR (10,000 cells/well), exposed to OVAb and co-cultured 1:1 with OT-I cells. Normalizing to WT BMDCs revealed the higher cross- presentation efficiency of DC^{2114} cells. N=3, triplicate wells. **g)** The strong OT-I proliferation induced by CpG-matured CD8α^{+} DC^{2114} exposed to OVAb could be easily discerned by microscopy as large Hoechst^{+} colonies (black staining). Bar = 400 µm. Error bars are means ± SEM, p *< 0.05, *** < 0.001 using a 2-way ANOVA and Sidak's post-test for panels d and e, and a Student's t-test for panels b and f.

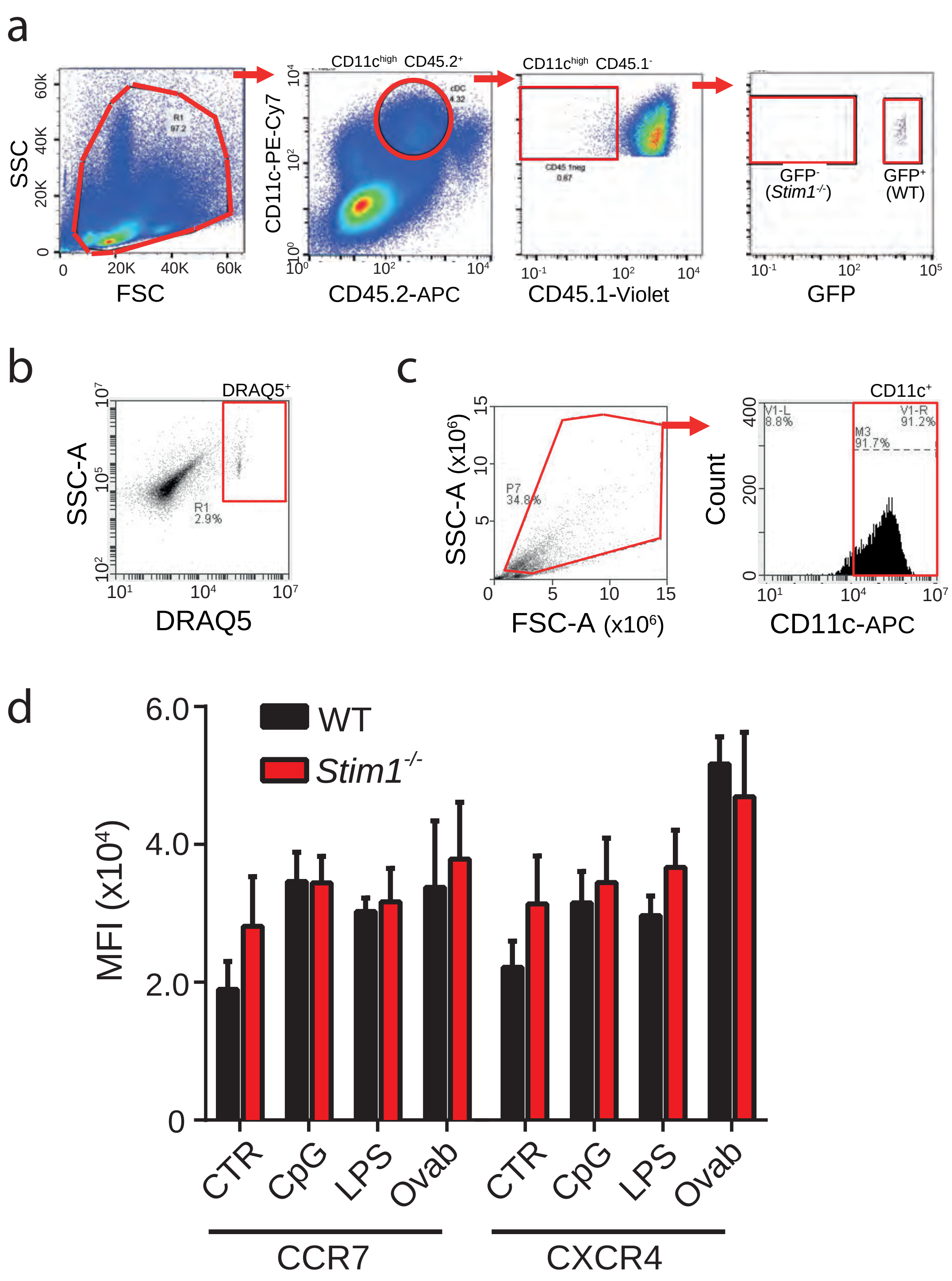

Supplementary Fig. 2. a) Full gating strategy for the *in vivo* migration assay in Fig. 2a. **b)** Full gating strategy for the *in vitro* migration assay in Fig. 2b. **c)** Full gating strategy for panel d, as well as for Figs 5b and 5d. **d)** Cell-surface immunostaining revealed no significant differences in CCR7 or CXCR4 receptors between WT and *Stim1$^{-/-}$* CD11c^{+} BMDCs in either controls or when cells were exposed to CpG, LPS (18 h) or OVAb (4 h). N=3, in triplicate wells. Error bars are means + SEM.

Supplementary Fig. 3

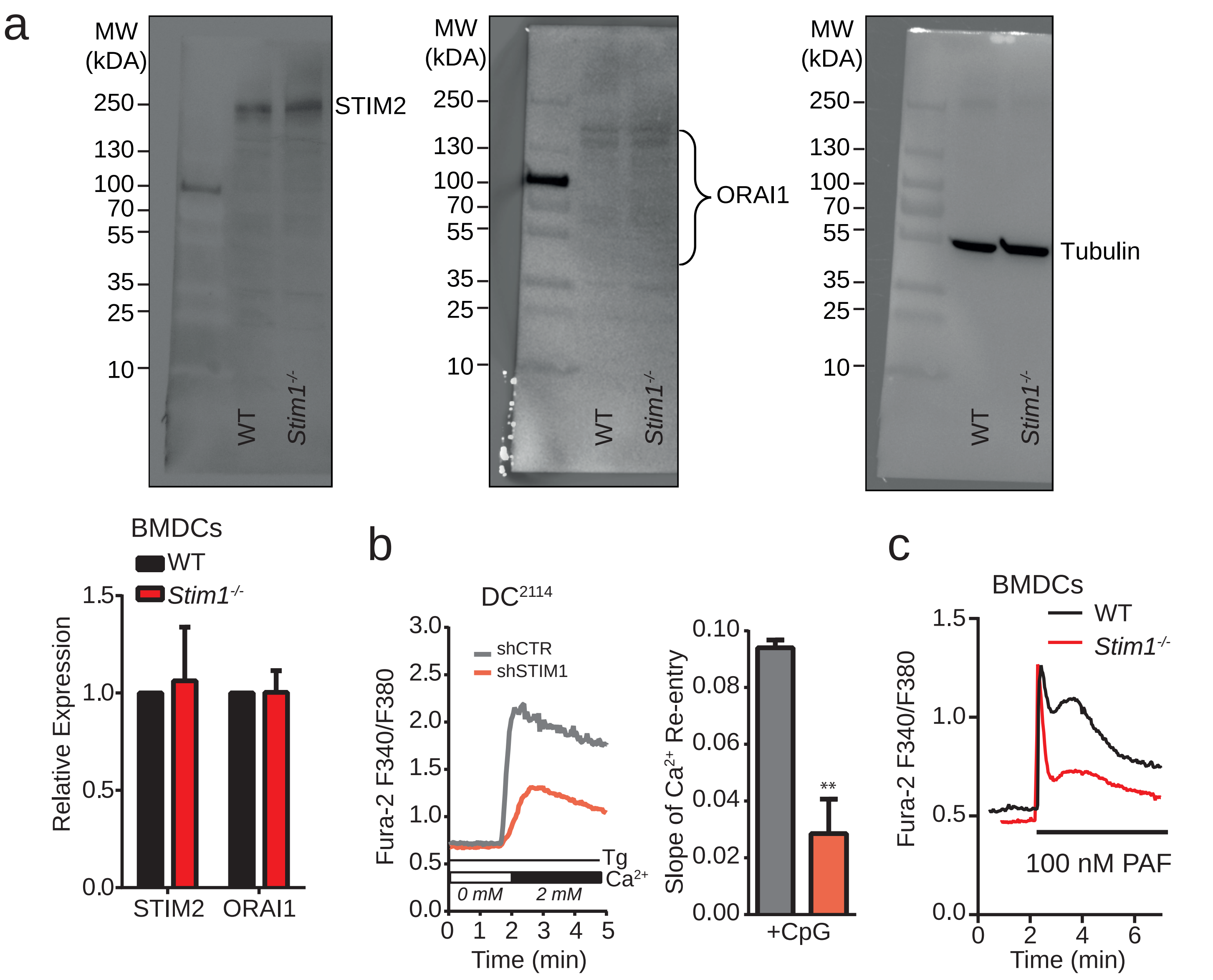

Supplementary Fig.3. a) Western blots showed no differences in STIM2 (left) or Orai1 (middle) expression in *Stim1*$^{-/-}$ BMDCs as compared to WT. N=3. Values are normalized to tubulin (right) expression. **b)** STIM1 knockdown strongly decreased SOCE, measured in CpG matured DC^{2114} cells as the slope of Ca^{2+}-re-entry after store depletion with 1 μM Tg in Ca^{2+}-free medium and 2 mM Ca^{2+} re-addition. N=3. **c)** Addition of 100 nM PAF produces similar Ca^{2+} responses as 2 μM PAF (See Fig. 3b). Error bars are means + SEM. p **< 0.01.

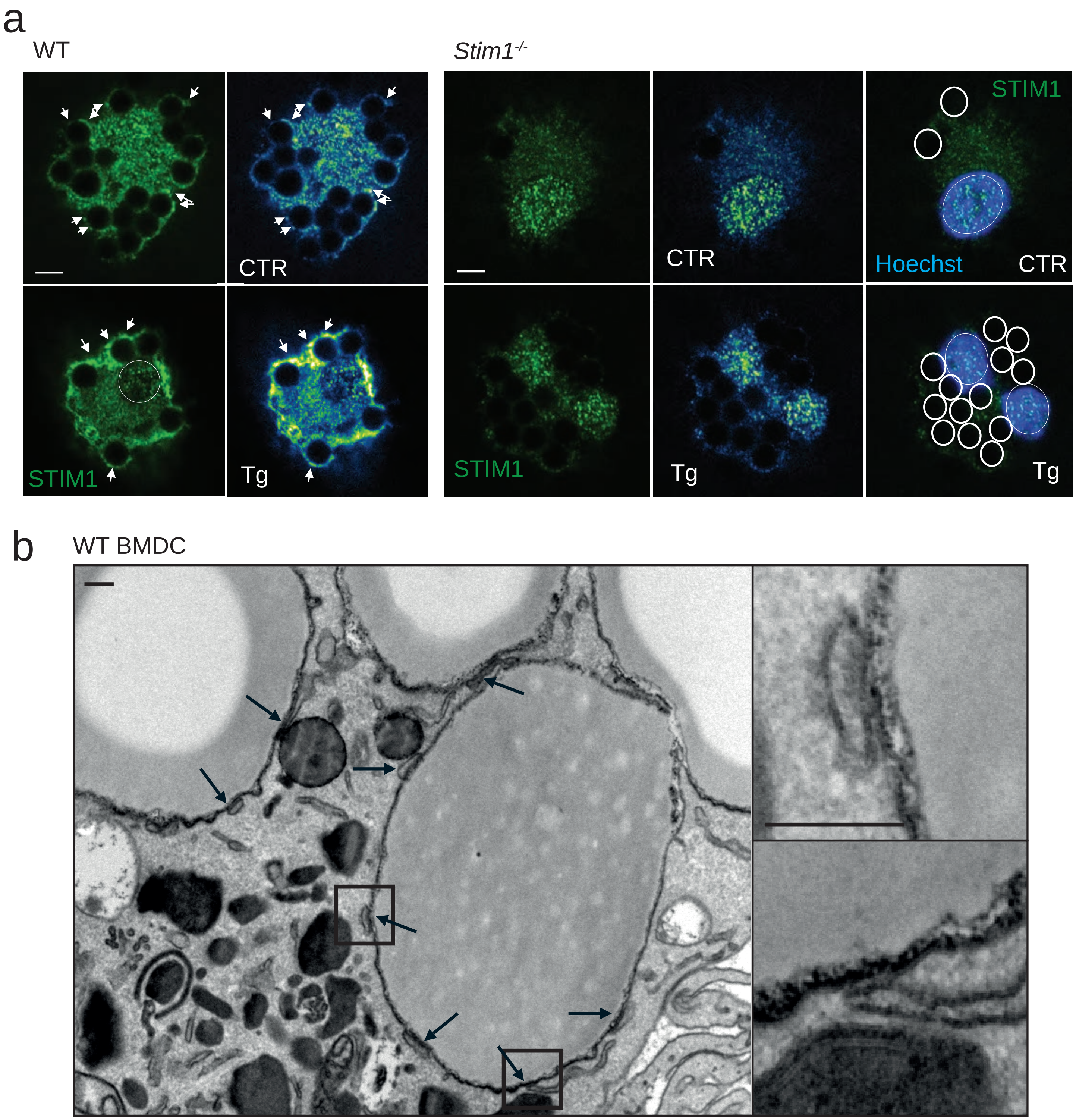

Supplementary Fig. 4. a) Immunofluorescent staining with mouse anti-STIM1 of WT (left) and *Stim1*$^{-/-}$ (right) BMDCs exposed to OVAb for 30 min and treated or not (CTR) with 1 μM thapsigargin (Tg) for the last 10 min before fixation. Small periphagosomal STIM1 clusters (arrows) can be discerned that are more prominent when cells are treated with Tg (lower left-hand panels). Images are shown using identical contrast settings, and pseudocolored images are shown to the right of their respective images to highlight higher-intensity clusters. A small background cytosolic staining and a prominent, non-specific nuclear staining are observed in STIM1-deficient cells. Phagosomes and nuclei are outlined in white in the right-hand panels for STIM1-deficient cells. White bar = 3 μm. **b)** Transmission electron micrograph of a WT BMDC exposed to OVAb for 30 min. Arrows and insets illustrate multiple sites of contact between the ER and phagosomes. Bar = 100 nm.

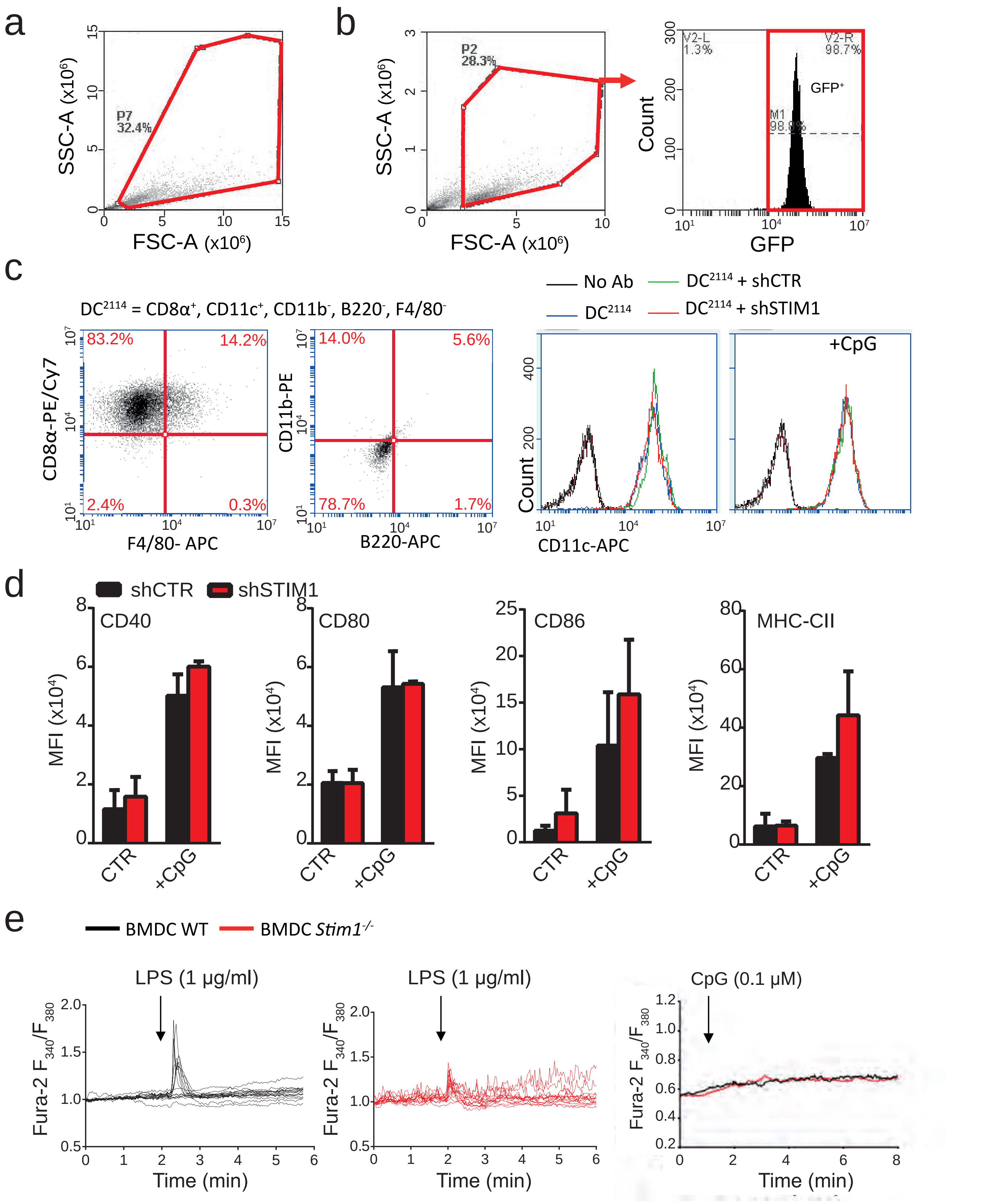

Supplementary Fig. 5. a) Gating strategy for Fig 5a. **b)** Full gating strategy for panels c and d. **c)** Representative dot plot (left) showing that DC2114 cells are CD8α+, F4/80-, CD11b- and B220-. Representative histograms (right) showing that CD11c levels are not affected by expression of either shCTR or shSTIM1 in either immature or CpG-matured DC2114 cells. **d)** Cell surface immunostainings showed no significant differences in maturation markers CD40, CD80, CD86 and MHC-II expression or upregulation upon pre-treatment with CpG. N=3. **e)** Representative single-cell traces from coverslips where a subset of WT and *Stim1*-/- BMDCs show an increase in cytosolic Ca^{2+}, detected using Fura-2 imaging, upon acute exposure to LPS (right, middle panels). No responses were detected in response to CpG (right panel, whole-coverslip averages are shown). Error bars are means + SEM.

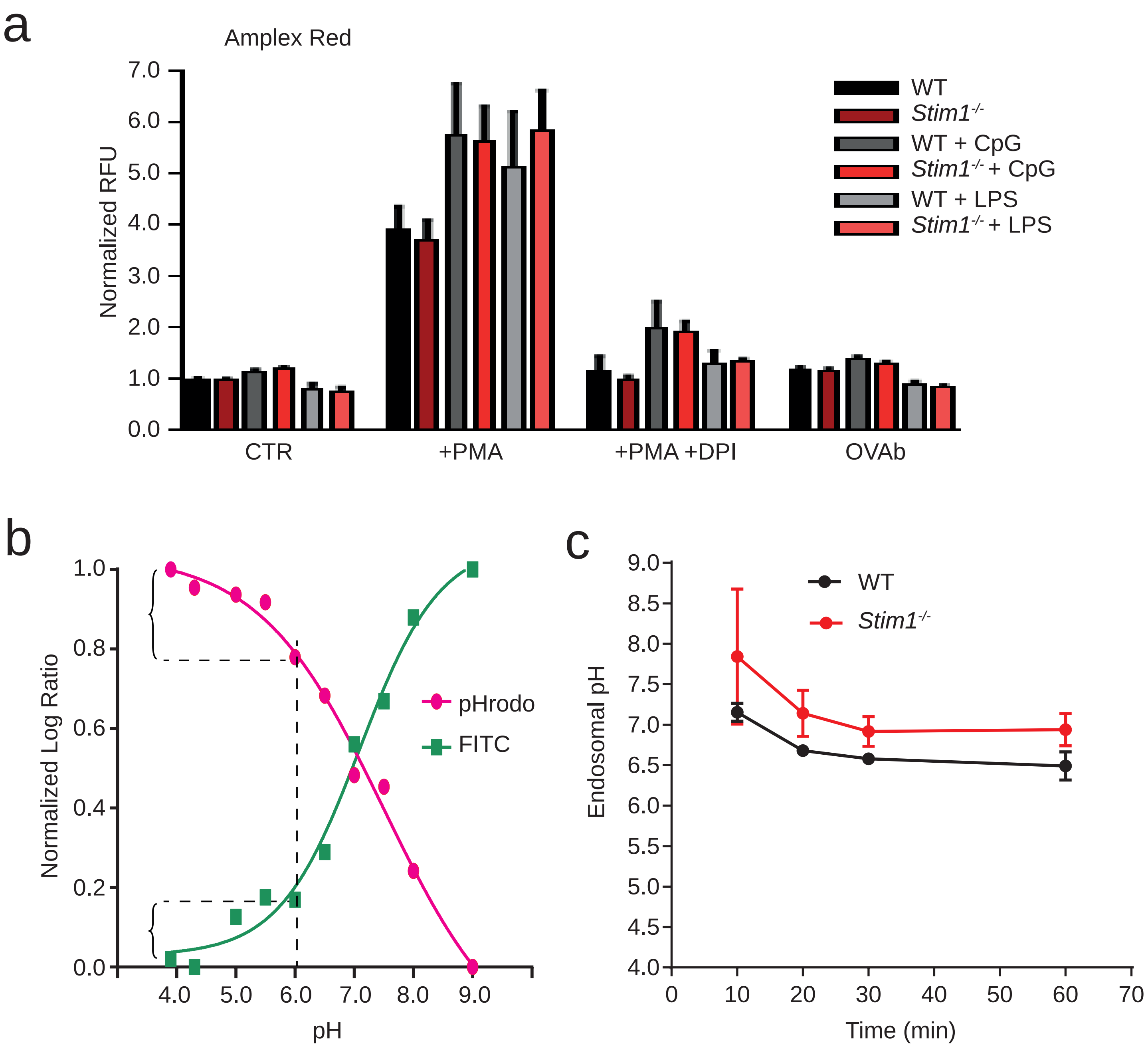

Supplementary Fig. 6. a) Extracellular ROS levels measured with an Amplex Ultra Red assay in immature or CpG- or LPS-matured WT and *Stim1*$^{-/-}$ BMDCs revealed no differences. Exposure to 100 nM PMA as a positive control increased extracellular ROS, an effect that was blocked by 10 µM DPI. In contrast to intracellular ROS (see Fig. 6a) OVAb did not induce detectable levels of extracellular ROS. N=3, triplicate wells. **b)** Representative *in situ* calibration curves for FITC- and pHrodo/Alexa568-coupled zymosan after 90 min of phagocytosis. The logs of the ratio values are normalized between 0 and 1 to allow comparison of the sensitivity of the two dyes on the same scale. pHrodo displays a higher sensitivity at lower (< 6.0) pH values than FITC. **c)** Endosomal pH was measured in BMDCs loaded with FITC- and Alexa-568-coupled dextran and analysed by flow cytometry. Although the endosomal pH of *Stim1*$^{-/-}$ BMDCs showed a trend for a higher pH, the difference was not significant. N = 5/4/5/5 for WT (10/20/30/60 min) and N=3 for all *Stim1*$^{-/-}$ timpoints. Error bars are means ± SEM.

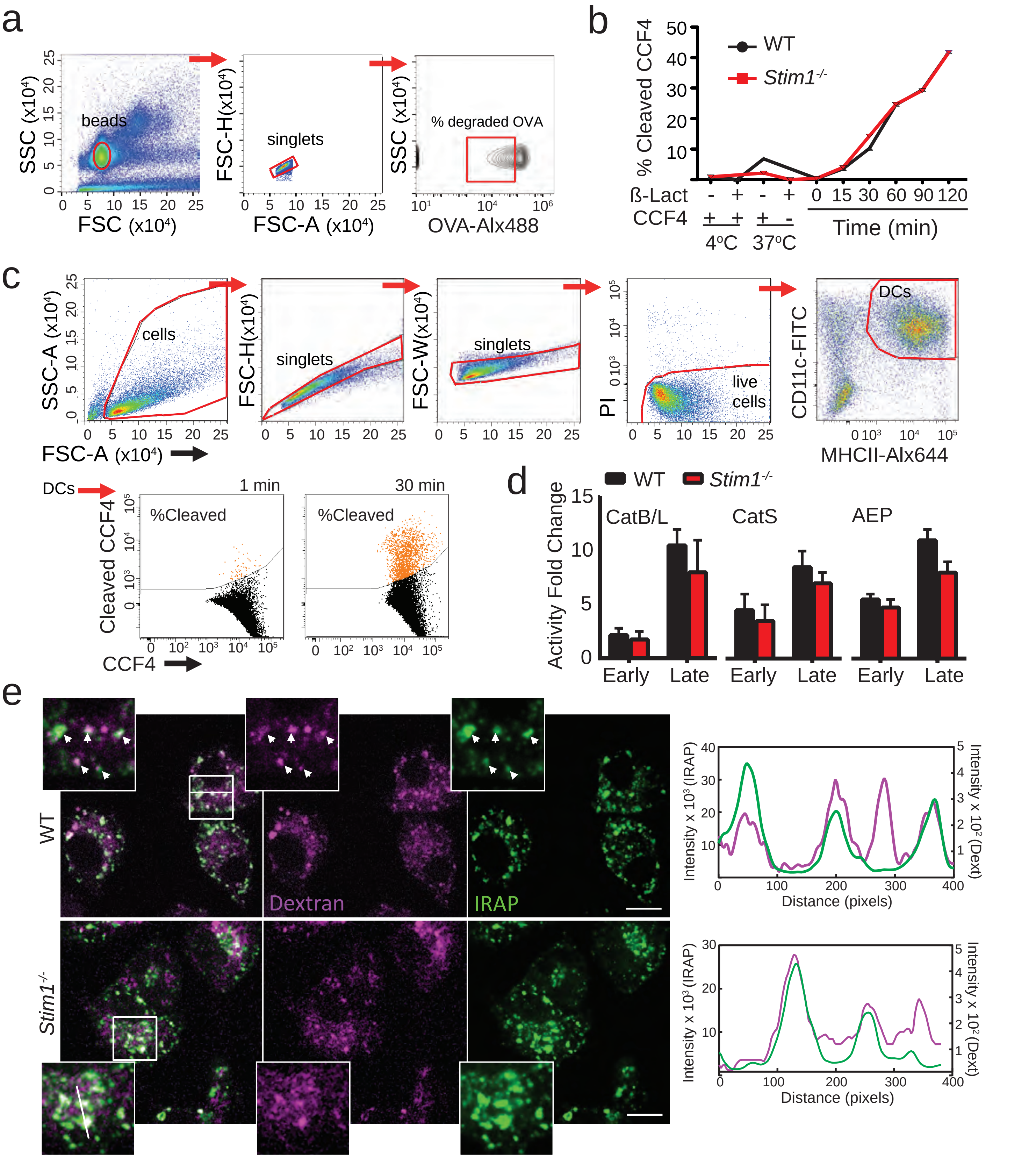

Supplementary Fig. 7. a) Full gating strategy for Fig. 7b. **b)** Cytosol transfer was measured by loading cells with CCF4 (4 µM) for 1h before incubating them with β-lactamase (β -lact) for the indicated time points. Negative controls cells incubated at 4ºC or in the absence of either enzyme or substrate are shown on the left. N=3. **c)** Full gating strategy for panel b. **d)** Protease activity was measured in early (30 min) and late (60 min) phagosomes. The activity of cathepsins B/L (Cat B/L, left), cathepsin S (CatS, middle) and asparagine endopeptidase (AEP, right) were not significantly different in STIM1-deficient BMDCs as compared to WT. N=3. Error bars are means ± SEM. **e)** Dextran-containing endosomes (magenta) partially overlap with IRAP-positive endosomes (green) in both WT and STIM1-deficient cells. Line-scan profiles illustrating signal overlap are shown on the right. Bar = 10 µm.
