# Supplementary material for: STIM1 promotes migration, phagosomal maturation and antigen cross-presentation in dendritic cells
Source: Nat Commun. 2017 Nov 24;8:1852. doi: 10.1038/s41467-017-01600-6 (PMC5701258; doi:10.1038/s41467-017-01600-6)
Supplement: Supplementary file 3 — Description of Additional Supplementary Files [file 41467_2017_1600_MOESM3_ESM.pdf]

Editorial Note: Parts of this peer review file have been redacted as indicated to maintain the confidentiality of unpublished data.

Reviewers' comments:

Reviewer #1 (Remarks to the Author):

Remarks to the author

This paper aims to demonstrate the involvement of STIM-1 in the antigen processing competences of murine dendritic cells in cross presentation. This question reports the fundamental aspects of the antigen processing mechanisms that might have huge therapeutic implications.
The experiments seem carefully done and the findings will be interesting. The updated analytical methods and appropriate animal models support the achieving of highest quality data that support this work. This paper is very interesting and provided intriguing new elements on the cross presentation mechanisms.
I have the following comments.

Introduction

First, the paper provides DCs as phagocytic cells, that is a bit excessive. It could be said that DCs have phagocytic skills different from those of macrophages (Manolova et al. 2008).

The discussion of a controversy over the involvement of respective STIM 1, STIM-L1 or STIM 2 has no place because many studies show that DC calcium homeostasis in mouse and human are different and are controlled by different forms of STIM.

Results

Numerous works on DC migration showed that this skill is depend on Ca2+ homeostasis. I do not understand what are the connections between the cross presentation and migration? What are your arguments to connect it? How could be explained the difference between 24h and 48h in the in vivo experiments (fig 2a)? Did the KO DCs migrated more slowly and finally arrived later? Please comment on this and update as appropriate.

Concerning the Ca2+ traces on the fig 2b, we cannot say that KO cells have a decrease for fMIFL, the trace is different (slope, rate) from this with WT cells but a Ca2+ entry is observed. It is not the case for SDF-1. With CCL21, there is no Ca2+ entry and a migration independent of Ca2+?? Please comment on this and update as appropriate.

The sentence in Line 113 is questionable because it is difficult to correlate mathematically these two types of experiments.

In SOCE measurements, the slope of the decrease could indicate where the Ca2+ is leaking. Calcium can go to the mitochondria or another vesicles. Do you have any idea about that? Is it possible to specifically labelled Ca2+ in the phagosome as for ER trackers? Please comment on this and update as appropriate.

The experiments showing the hotspots of Ca2+ are really exciting. Is it possible to co-localise STIM molecule and Ca2+ hotspots in WT mice? The fig3d does not convincing me about the localisation of STIM. Please comment on this and update as appropriate.

STIM could be on the plasma membrane associated with several other partners than ORAI 1 such as ORAI-3 to form ARC (Thompson et al. 2013) or TRPC1. The localisation of STIM-1 is essential to the demonstration. Please comment on this and update as appropriate.

This work is really very close to demonstrate the role of STIM in DC cross-presentation but for now it shows only that cytosolic changes in calcium homeostasis have an impact on the organization and migration of intracellular organelles. It needs to be more specific about the location of STIM on phagosomes or other membrane and the Ca2+ motion. The general impression is that the deficit in cross-presentation could be due to a defective migration of the involved vesicles. Please comment on this and update as appropriate.

Minor remarks
− The H-2 of DC2114 cell line (in vitro assay of cross presentation) must be done in M&M section.
− Regarding the in vitro migration showed in fig2., the percentage of control cells that have migrated must be done in the legend of the figure. We know that under 25% of migration of control cells, experiments are not relevant.

− The cell line is not clearly indicated on the fig3b.

− PAF was used to stimulate DC cell line (fig3b). The IC50 of PAF is 10 nM, the 2μM of PAF is a huge concentration. Did you obtain the same effect with only 100 nM?

− Have you paid attention that some trackers have a various Kd for Ca2+ according to the pH? Please comment on this and update as appropriate

Reviewer #2 (Remarks to the Author):

In their report „STIM1 promotes migration, phagosomal maturation and antigen cross-presentation
in dendritic cells", Nunes-Hasler et al. thoroughly investigate the contribution of store-operated Ca2+ entry, specifically the role of STIM1 by myeloid specific deletion of STIM1, for different physiological functions of dendritic cells. While a number of reports using a variety of cell lines have described an influence of STIM1 ablation for cell migration, the interesting and novel finding of this report concerns the defects seen in cross-presentation, phagosomal proteolysis and phago-lysosome fusion. Here the detailed anatomical localization of contact sites between the ER and the phagosomes is interesting and beautifully done and the decrease seen in the contact sites in the STIM1 deficient cells hint towards a novel function of STIM1. However, regulation of ER-phagosomal contact sites by STIM1 was also reported in the author's earlier paper (Nunes et al, Current Biology 2012) using 2D EM pictures. Very interesting and novel are the pH measurements of different phagosomes within a single cell, and it would be nice to discuss these in relation to the result seen byo Tlili, Dupré-Crochet et al, 2011. A disparate and surprising result is that ROS production is not affected by STIM1 deletion (see comment 4).

Previously, the authors used a similar genetic approach to delete the Stim1 gene in neutrophils from C57BL/6 mice (Stim1fl/fl; LysMCre/+). Here Stim1 ablation decreased phagocytosis in neutrophils by 50% and caused a corresponding 50% decrease in the frequency of the local Ca2+ elevations occurring near phagosomes (Nunes et al., 2012). A more thorough discussion of possible differences between DC and neutrophils would be commendable.

Major concerns
1. Figure 1: Cross-presentation is Ca dependent but strongest effect seen upon inhibition of NOX2

with DPI? Fig 1f: Images of the differences seen upon Hoechst staining are not very convincing and appear dependent on the chosen image window, i.e. the shSTIM1 image shows large clusters at the right edge of the image. How was the initial seeding of the colonies controlled?

2. Figure 2: What is the explanation for the recovery of %CD45.2 positive cells after 48h? In Figure 1 DL and NDL lymph nodes were harvested after 72 h, so how can the reduced migration contribute to the defective cross-presentation in vivo?

3. Figure 4 and lines 167: The interpretation of Figure 4c should be more careful: BAPTA does not reduce the relative MFI by 50% when comparing the 4h values and especially not after LPS stimulation (does LPS lead to significant ingestion?) and why were BAPTA and La3+ not applied for 24h to compare to the 24h values?

4. Figure 5: Were the CpG matured cells treated similar to the cells in Figure 1c concerning the T:DC ratios? Did the authors consider using other physiological stimuli such as fMLF to investigate the SOCE dependent ROS production as done by other authors (i.e. Brechard et al, Saul et al)? It is clear that with very strong stimuli such as PMA or Zymogen a contribution of SOCE is overridden. Does 2APB block OVAb dependent ROS production? The same reasoning applies to the pH measurements. Here, due to technical reasons, only OVA-zymogen beads were used. Is it possible that different stimuli use different intracellular pathways to produce ROS (i.e. SOCE dependent vs TRPM2 dependent)?

5. The analysis concerning the phago-lysosomal fusion in Figure 6 is very interesting. One aspect that is not entirely clear is how and if loading with BAPTA-AM would or could affect the Ca2+ content within the lysosomes or phagosomes. If STIM1 presence leads to activation of Orai on the phagosomal membranes as proposed by the authors in earlier work (Nunes et al 2012), deletion of STIM1 would likely increase phagosomal Ca2+ content, whereas BAPTA AM in the cytosol or in the lumen of the phagosome would decrease it. Also the % OVA degradation is significantly different only in the first 30-60 min whereas the P-L fusion Index is affected by STIM1 only at 90 min. Even if STIM1 function is only structural, BAPTA AM would lead to an increased clustering and increased interaction sites between ER and phagosomes (Fig. 3). Analysis of the contact sites (as in Fig. 3) in the presence of BAPTA AM might clarify this.

Minor:
Line 32: of which there are two paralogs (STIM1 and STIM2) each of which show additional splice variants
Line 35: Please include references for the remodeling role of STIM proteins in ER junction formation

Reviewer #3 (Remarks to the Author):

This manuscript reports that STIM1 is required for optimal dendritic cell (DC) migration, phagosomal maturation and, most importantly, for antigen cross-presentation. Using both in vivo and in vitro measurements, the authors report a marked diminution of antigen cross-presentation efficiency. They propose that impaired chemotaxis and antigen proteolysis in phagosomes account for these observations.

Despite the considerable number of sophisticated approaches applied, it was unclear to me what mechanism is proposed to account for the reduced cross presentation. On one hand, the authors report reduced contacts between the ER and phagosomes in cells lacking STIM1. They also find that calcium hotspots around the phagosome are reduced, which may account for the reduced fusion and maturation. These observations, however, generate a number of questions and issues

that are critical and, in my view, have not been resolved in the current version. These include:

1) STIM1 associates with the plasma membrane when the luminal calcium of the ER is depleted, but is otherwise not attached to it. Do the authors envisage a similar process connecting the ER to phagosomes via STIM1? If so, why is STIM1 activated during phagosome maturation? Is the calcium in the ER depleted at the time when they analyzed the ER contacts (the time should be specified in the text and figure legend). If so, this should be demonstrated and the underlying mechanism should be defined. If not depleted of calcium, why would the ER associate with phagosomes in a STIM1-dependent manner? Is this a novel mode of function of STIM1 and, if so, is it unprecedented?
2) If the mechanism of action of STIM1 involves calcium release from phagosomes, what activates STIM1 and, as importantly, what is the evidence that SOCE or TRPC channels exist and are functional in the maturing phagosome? Would the internal acidification be compatible with their activity? Late endosomes/lysosomes contain TRP-ML1 but, to my knowledge, this channel is not activated by STIM1.
3) The protease content of phagosomes of WT and STIM1-KO cells was found to be indistinguishable. This seems incompatible with the finding that lysosome fusion is inhibited. The authors postulate that perhaps other proteases not investigated are responsible for the observed differences. However, differential delivery of another protease would require that the putative protease be selectively localized in a subpopulation of lysosomes. What is the evidence that this can occur?
4) In the end, it is unclear whether more proteolysis favors cross-presentation, or whether the opposite is true. The notion that reduced phagosomal acidification promotes cross-presentation as a result of reduced proteolysis seems incompatible with the interpretation of this paper.
5) Along the same lines: is the very acidic pH reported in the majority of the phagosomes consistent with the results of Amigorena's group? Is it compatible with effective cross-presentation? Along the same lines, the companion manuscript of Manoury et al does report changes in phagosomal pH, in contrast to the result in the present paper. What account for this apparent discrepancy? Does this imply that UNC93 is having effects other than through STIM1?
6) The large difference between the pH values reported using two different methods needs to resolved. Which one is right?
7) Were similar concentrations of chemoattractant used to measure calcium and chemotaxis? If not, the conclusions need to be tempered down accordingly.
8) What is the evidence that the calcium hotspots originate from the phagosome? If that is not the correct interpretation, what is their source (ER? Lysosomes?) and their relevance to the fusion process? Are these sustained events and, if so, why is the phagosomal calcium not soon depleted?
9) What is the relevance of the inhibitory effect of DPI and of concanamycin A to calcium?
10) In Figure 2 the authors quantify calcium transients. However these presumably reflect mostly calcium release from the ER. How are they relevant to STIM function?
11) Is CCL21 not more relevant to cross-presentation in situ than is fMIFL? If so, is the explanation offered adequate?
12) Why does OVAb stimulate the NADPH oxidase?
13) Is cell-associated bead fluorescence an adequate measure of phagocytosis? Are there no adherent beads attached extracellularly or have they been ruled out? Please explain.

The comments of the reviewers are in *italics*, our response to these comments in plain letter

Reviewers' comments:

Reviewer #1 (Remarks to the Author):

Remarks to the author

This paper aims to demonstrate the involvement of STIM-1 in the antigen processing competences of murine dendritic cells in cross presentation. This question reports the fundamental aspects of the antigen processing mechanisms that might have huge therapeutic implications.
The experiments seem carefully done and the findings will be interesting. The updated analytical methods and appropriate animal models support the achieving of highest quality data that support this work. This paper is very interesting and provided intriguing new elements on the cross presentation mechanisms.
I have the following comments.

We thank the reviewer for the positive comments.

Introduction

1)First, the paper provides DCs as phagocytic cells, that is a bit excessive. It could be said that DCs have phagocytic skills different from those of macrophages (Manolova et al. 2008).

We agree that the phagocytic process differs in macrophages and dendritic cells, particularly with respect to the size of the particle ingested and the fate of the phagocytosed material. We have modified the introduction to include the cited reference and more clearly highlight this point.

2)The discussion of a controversy over the involvement of respective STIM 1, STIM-L1 or STIM 2 has no place because many studies show that DC calcium homeostasis in mouse and human are different and are controlled by different forms of STIM.

Agreed. We have modified the introduction to more clearly state that the differences in isoform requirement found are likely related to species-specific differences

Results

3)Numerous works on DC migration showed that this skill is depend on Ca2+ homeostasis. I do not understand what are the connections between the cross presentation and migration? What are your arguments to connect it?

Cross-presentation of antigens captured by DC to lymphocytes occurs in lymph nodes. A migration defect could thus contribute to the reduced cross-presentation *in vivo* as DCs must migrate to the site of injection and then to lymph nodes after ingesting ovalbumin beads injected in the dermis. This is why we analysed the migration defects in STIM1 depleted cells and the contribution of STIM1 to the associated calcium signals. We have added a sentence to clarify this point. See also our response to reviewer 2, point #2.

4)How could be explained the difference between 24h and 48h in the in vivo experiments (fig 2a)? Did the KO DCs migrated more slowly and finally arrived later? Please comment on this and update as appropriate.

Indeed we believe that STIM1-deficient DCs migrate more slowly in tissues as suggested by our in vitro migration data (Fig 2c). A defective migrational guidance might also contribute, as shown for ORAI1-deficient neutrophils [1]. This translates into a reduced amount of cells recruited to lymph nodes at 24h, an effect that disappears at 48h as STIM1-deficient DC slowly accumulate in lymph nodes. These points are now discussed. See also our response to reviewer 2, point #2.

5)Concerning the Ca2+ traces on the fig 2b, we cannot say that KO cells have a decrease for fMIFL, the trace is different (slope, rate) from this with WT cells but a Ca2+ entry is observed.

Ca^{2+} responses evoked by InsP3-generating agonists like fMIFL comprise two components, an initial rapid elevation caused by the release of Ca^{2+} from the ER and a delayed elevation reflecting the entry of Ca^{2+} across membrane channels. When Ca^{2+} entry is prevented pharmacologically, stereotypical Ca^{2+} transients with a sharp initial rise followed by a rapid decay are observed that are identical to the residual Ca^{2+} transients of STIM1 KO cells. These transients reflect Ca^{2+} release from stores and the lack of the delayed elevation is indicative of a defect in SOCE. Comparison of the area under the curve of the evoked responses, a parameter that integrates the release and influx components, indicates that STIM1-mediated influx contributes ~50% of the Ca^{2+} signal. We have added a sentence in the results to clarify this point.

6)It is not the case for SDF-1.

SDF-1 mobilizes Ca^{2+} less efficiently than fMIFL and the resulting Ca^{2+} elevations are not clearly biphasic as the Ca^{2+} release component is less explosive and of reduced magnitude. As a result, the release and influx component are not as easily resolved than with fMIVIL. Nevertheless, quantifying the area under the curve allows to estimate the contribution of STIM1-mediated influx to be again ~50% of the Ca^{2+} signal.

7)With CCL21, there is no Ca2+ entry and a migration independent of Ca2+?? Please comment on this and update as appropriate.

Indeed this was a surprising result as CCL21 was reported to be a Ca^{2+}-mobilizing agonist [2] yet other studies have shown that CCR7 is only coupled to Ca^{2+} signals if DCs are matured in the presence of prostaglandin-2, indicating that Ca^{2+} signals are not an obligatory downstream element of CCR7 engagement. [3]. We tested several doses of CCL21 but never observed a Ca^{2+} response, despite the effectiveness of this cytokine as chemoattractant. We can only speculate as to why this is, but the preserved migration indicates that the cytokine was bioactive in our hands. One possibility is that, similar to the TLR4 and CD14 receptors for LPS, there is a co-receptor that links CCR7 signalling to Ca^{2+} that is only activated under certain conditions. Parallel signalling pathways (such as phosphorylation) may be sufficient to trigger migration in the absence of Ca^{2+} signals and this putative co-receptor was not engaged in our experimental conditions. Alternatively, impurities in the preparation of

recombinant CCL21 could have generated Ca^{2+} signals in previous studies.

8)The sentence in Line 113 is questionable because it is difficult to correlate mathematically these two types of experiments.

We have clarified the wording of this sentence.

9)In SOCE measurements, the slope of the decrease could indicate where the Ca2+ is leaking. Calcium can go to the mitochondria or another vesicles. Do you have any idea about that?

The outcome of the Ca^{2+} entering across SOCE channels is an interesting question. In the presence of SERCA inhibitors, cytosolic Ca^{2+} ions cannot be sequestered in the ER and are extruded from the cell by PMCA or NCX exchangers. These Ca^{2+} extrusion mechanisms are very efficient and, together with the fast and slow Ca^{2+}-dependent inhibition of ORAI channels, contribute to the decay of the Ca^{2+} elevation observed in cells treated with thapsigargin. A fraction of the entering Ca^{2+} ions can be taken up by other intracellular organelles. Mitochondrial Ca^{2+} uptake was shown to be minimal during SOCE due to the low Ca^{2+} affinity of the MCU complex [4]. Mitochondria are therefore likely not a major determinant in the kinetics of the Tg-induced response. However we cannot rule out uptake by lysosomes or endosomes, mechanisms which are less well understood. Since many factors can influence the decaying phase of the Tg-induced response we believe that it would be difficult to derive firm conclusions from this parameter and limit our analysis to the slope of the increase which in the absence of SERCA pumping is a reliable indicator of SOCE.

10)Is it possible to specifically labelled Ca2+ in the phagosome as for ER trackers? Please comment on this and update as appropriate.

[Redacted]

The experiments showing the hotspots of Ca2+ are really exciting. Is it possible to co-localise STIM molecule and Ca2+ hotspots in WT mice?

We have now performed this experiment and show that STIM-mCherry clusters colocalize with Ca^{2+} hotspots around phagosomes in BMDCs (see new Fig 3d). In response to reviewer 3 comments we also show that pharmacological inhibition of SOCE channels and of InsP3 Ca^{2+} release channels reduces the occurrence of periphagosomal Ca^{2+}microdomains (new Fig 3c).

The fig3d does not convincing me about the localisation of STIM. Please comment on this and update as appropriate. STIM could be on the plasma membrane associated with several other partners than ORAI 1 such as ORAI-3 to form ARC (Thompson et al. 2013) or TRPC1. The localisation of STIM-1 is essential to the demonstration. Please comment on this and update as appropriate.

Transfection and transduction of BMDCs with fluorescent STIM1 is very inefficient (less than 1% of fluorescent cells), and cells that are successfully transduced show very low levels of fluorescence, hence the quality of the images we show are admittedly low. We have repeated this experiment and again observed a scattered punctate STIM1 signal lacking the reticular pattern observed in other cell lines (see new Fig 3d). However mCherry-STIM1

clusters were observed around phagosomes when cells are phagocytosing (new Fig 3d). In addition we have now performed immunofluorescence staining of endogenous STIM1 in DCs (see new supplementary Fig S4). The staining pattern resembles the punctate morphology seen in transfected cells and the periphagosomal STIM1 staining was more prominent when we stimulated the cells with thapsigargin.

This work is really very close to demonstrate the role of STIM in DC cross-presentation but for now it shows only that cytosolic changes in calcium homeostasis have an impact on the organization and migration of intracellular organelles. It needs to be more specific about the location of STIM on phagosomes or other membrane and the Ca2+ motion. The general impression is that the deficit in cross-presentation could be due to a defective migration of the involved vesicles. Please comment on this and update as appropriate.

Indeed, since both global as well as local Ca^{2+} defects are observed in STIM1-deficient cells we cannot conclude definitively that the abortive local Ca^{2+} signals around phagosomes are the ones that lead to cross-presentation defects. To strengthen this link, we now demonstrate that STIM1 is recruited to DC phagosomes, where it colocalizes with the Ca^{2+} hotspots. The reduced periphagosomal Ca^{2+} signals, in turn, correlate with a reduced delivery of vesicles to phagosomes, a process known to be Ca^{2+}-dependent. In addition, we now report an endosome fusion defect and a decreased activity of the endosomal peptidase IRAP in STIM1 deficient cells (Fig 7d and 7e). We acknowledge however that we can only state that we can correlate the occurrence of these defects with reduced cross-presentation and have modified our discussion accordingly.

Minor remarks
− The H-2 of DC2114 cell line (in vitro assay of cross presentation) must be done in M&M section.
This information has now been added.

− Regarding the in vitro migration showed in fig2., the percentage of control cells that have migrated must be done in the legend of the figure. We know that under 25% of migration of control cells, experiments are not relevant.

We now show these data as percentage of migrated cells and have clarified this point in the figure legend (Fig 2). No significant differences in baseline levels were detected between WT and STIM1 cells and this information is now stated in the results.

− The cell line is not clearly indicated on the fig3b.

- The cells used (BMDC) are now mentioned in the figure.

− PAF was used to stimulate DC cell line (fig3b). The IC50 of PAF is 10 nM, the 2µM of PAF is a huge concentration. Did you obtain the same effect with only 100 nM?

Yes. 2uM is a supramaximal concentration that we normally use for stimulating cell lines, in order to obtain a maximal SOCE response. We have repeated the experiments using only 100 nM PAF and obtained the same results. (see Fig S3d)

− Have you paid attention that some trackers have a various Kd for Ca2+ according to the pH? Please comment on this and update as appropriate

Yes. Most BAPTA-based calcium dyes including Fura-2 are pH sensitive and have a reduced calcium affinity at pH below 6. Fluo-8 is derived from fluorescein and might be similarly quenched by acidic pH. This is a concern for phagosomal calcium measurements, as discussed above. However, the cytosolic pH does not fall below pH 6 during calcium elevations (See Poburko JBC 2011[6]) and pH variations are therefore not expected to impact our measurements of the global and local cytosolic calcium signals.

Reviewer #2 (Remarks to the Author):

In their report „STIM1 promotes migration, phagosomal maturation and antigen cross-presentation in dendritic cells", Nunes-Hasler et al. thoroughly investigate the contribution of store-operated Ca2+ entry, specifically the role of STIM1 by myeloid specific deletion of STIM1, for different physiological functions of dendritic cells. While a number of reports using a variety of cell lines have described an influence of STIM1 ablation for cell migration, the interesting and novel finding of this report concerns the defects seen in cross-presentation, phagosomal proteolysis and phago-lysosome fusion. Here the detailed anatomical localization of contact sites between the ER and the phagosomes is interesting and beautifully done and the decrease seen in the contact sites in the STIM1 deficient cells hint towards a novel function of STIM1. However, regulation of ER-phagosomal contact sites by STIM1 was also reported in the author's earlier paper (Nunes et al, Current Biology 2012) using 2D EM pictures. Very interesting and novel are the pH measurements of different phagosomes within a single cell, and it would be nice to discuss these in relation to the result seen byo Tlili, Dupré-Crochet et al, 2011.

We appreciate the reviewer's positive comments. Indeed we believe that resolving the chemistry of individual phagosomes by microscopy allows a better understanding of the phagocytic process. Substantial heterogeneity among neutrophils phagosomes was reported for ROS production by Tlili et al using DCFH2-yeast particles [7], and we also observed a broad distribution of phagosomal ROS and pH in neutrophils [8]. The two parameters are linked and phagosomes alkalinisation depends on the amount of ROS produced as initially reported by the Amigorena's group [9]. The pH heterogeneity of DC phagosome was much broader, which as discussed in the response to reviewer #3, point 6, complicates the measurements of average pH but renders DC able to present some peptides efficiently while destroying others. A short discussion on the implication of phagosomal ROS and pH heterogeneity is now added on pg. 14.

A disparate and surprising result is that ROS production is not affected by STIM1 deletion (see comment 4).

We were also expecting that STIM1 deficiency would decrease the activity of the NADPH oxidase and impair ROS production, as previously shown for neutrophils [10]. However, our data clearly indicate that this is not the case, see also point 4 below.

Previously, the authors used a similar genetic approach to delete the Stim1 gene in neutrophils from C57BL/6 mice (Stim1fl/fl; LysMCre/+). Here Stim1 ablation decreased phagocytosis in neutrophils by 50% and caused a corresponding 50% decrease in the frequency of the local Ca2+ elevations occurring near phagosomes (Nunes et al., 2012). A more thorough discussion of possible differences between DC and neutrophils would be commendable.

We agree that the differences are striking and have expanded the discussion to discuss this point. One remarkable difference is that compared to neutrophils DC exhibit 5-fold less periphagosomal Ca^{2+} microdomains despite a 5 fold increase in ER-phagosomes contacts. This suggests that ER contacts may play roles beyond calcium signalling in dendritic cells.

Major concerns
1. Figure 1: Cross-presentation is Ca dependent but strongest effect seen upon inhibition of NOX2 with DPI?

We used DPI and ConcA as positive controls to validate our assays, as these compounds inhibit cross-presentation by disrupting phagosomal ROS and pH homeostasis. In truth we were initially expecting changes in phagosomal ROS and pH to mediate the effects of STIM1 deficiency, but our data did not support this hypothesis.

Fig 1f: Images of the differences seen upon Hoechst staining are not very convincing and appear dependent on the chosen image window, i.e. the shSTIM1 image shows large clusters at the right edge of the image. How was the initial seeding of the colonies controlled?

Dc2114 cells were seeded at 10,000 cells per well and their even spreading at a confluency of 10-20% was verified visually by light microscopy. The DCs were irradiated so the cells observed at the low magnification shown are largely the T cell colonies. We found it interesting that in contrast to BMDCs which produced very small colonies, difficult to see at 10X magnification, the DC2114 colonies were huge. Indeed we noticed that shSTIM1 colonies were sometimes larger, but were consistently in smaller numbers. We tried to quantify this difference but the segmentation was inconsistent since the plastic bottom of the wells was not ideal for microscopy. We thus simply chose representative images to illustrate the differences found using chemiluminescence of BrDU and show the large colonies formed by DC2114. We have now moved this panel to the supplementary materials (Fig S1f).

2. Figure 2: What is the explanation for the recovery of %CD45.2 positive cells after 48h? In Figure 1 DL and NDL lymph nodes were harvested after 72 h, so how can the reduced migration contribute to the defective cross-presentation in vivo?

Initially DCs are recruited to the site of injection by DAMPs, PAMPs, or cytokines released at the site by inflammatory cells. Attracted DCs will phagocytose the foreign material that they encounter, and will enter the lymphatic system and migrate to the nearest draining lymph node. Our *in vitro* migration analysis indicates that the migration defect is selective for Ca^{2+}-dependent stimuli, but chemoattractant receptors that bypass the requirement for Ca^{2+} signals will eventually attract DCs to the lymph node. Indeed intact CCL21-dependent migration likely plays a role (see also our responses to reviewer 1' point 4 and reviewer 3's comment #11). Ca^{2+} signalling allows DCs to respond to a wider range of chemoattractants released at the infection sites and expressed by lymphatic vessels, enabling them to migrate more efficiently in tissues. STIM1-deficient DCs respond less efficiently to these signals and will migrate more slowly to the injection site and back to lymph nodes. A defective migrational guidance might also contribute, as shown for ORAI1-deficient neutrophils [1]. The migration defect translates into a reduced amount of cells recruited to lymph nodes at 24h, an effect that disappears at 48h as STIM1-deficient DC slowly accumulate in lymph nodes, attracted by Ca^{2+}-independent chemokines like CCL-21.

Once in the lymph node, DCs must interact with many different resident T cells in search of one bearing a matching TCR, a process that takes several hours. Although we observed an equal number of WT and STIM1-deficient DC at 48h, slowly moving cells arriving in the lymph node at 48h might not have sufficient time to make a successful immunological synapse and induce T cells to proliferate by 72 hrs. We thus conservatively conclude that the migration defect can potentially also contribute to the reduced cross-presentation that we observe in vivo. We have amended our discussion accordingly.

3. Figure 4 and lines 167: The interpretation of Figure 4c should be more careful: BAPTA does not reduce the relative MFI by 50% when comparing the 4h values and especially not after LPS stimulation (does LPS lead to significant ingestion?) and why were BAPTA and La3+ not applied for 24h to compare to the 24h values?

We have now specified the phagocytosis defects in the results section, and now highlight that in mature cells the defect observed with BAPTA was even smaller (or not significant, in the case of CpG). Indeed, we did see robust phagocytosis after overnight LPS stimulation (verified by microscopy), despite these levels being reduced as compared to non-stimulated controls. It is true that LPS stimulation is generally believed to suppress phagocytosis (Sallusto and Lanzavecchia, 1994[11]) but recent studies show that depending on the receptors engaged phagocytosis is only partially reduced or not reduced at all (Platt et al 2010 [12]). Indeed, the Amigorena group recently published only a 30-40% decrease at 90 min in BMDCs ([13], Fig S2 [13]), which is similar to what we observe here. We applied BAPTA and La^{3+} at 4 hrs only because we used this time point for our in vitro cross-presentation assays. We tested 24 hrs to be sure that we were not missing a hidden phagocytosis defect that accumulated over time, which was not the case.

4. Figure 5: Were the CpG matured cells treated similar to the cells in Figure 1c concerning the T:DC ratios?

In figure 5 we did not expose the DCs to T cells. Perhaps the reviewer meant the beads or zymosan to DC ratio? In this case, they were the same, always applied at a 20:1 ratio. This is now specified in the figure legends.

Did the authors consider using other physiological stimuli such as fMLF to investigate the SOCE dependent ROS production as done by other authors (i.e. Brechard et al, Saul et al)? It is clear that with very strong stimuli such as PMA or Zymogen a contribution of SOCE is overridden. Does 2APB block OVAb dependent ROS production?

DCs produce much lower levels of ROS than neutrophils or HL60 cells and we failed to detect a significant ROS production in response to fMLF, at both 100nM and 1 uM, regardless of the maturation state of the cells. We have measured the OVA-dependent ROS production in the presence of the SOCE inhibitor GSK7975A, reported to be more specific for ORAI proteins than 2-APB. We observed a slight but not significant increase in ROS production in all conditions. This confirms that SOCE inhibition does not impair ROS production by DC but given the limited gain of knowledge we did not include these pharmacological data in the revised manuscript.

The same reasoning applies to the pH measurements. Here, due to technical reasons, only OVA-zymogen beads were used. Is it possible that different stimuli use different intracellular

pathways to produce ROS (i.e. SOCE dependent vs TRPM2 dependent)?

Absolutely. We are not ruling out that other stimuli that engage different signalling pathway could evoke ROS production as we only rule out STIM1-dependent ROS production in our animal and cellular models. Ca^{2+}-dependent ROS production could persist as TRPM2 and TRPM4 have been shown to mediate Ca^{2+} signals in DCs, and residual STIM2-dependent signals may also play a role.

5. The analysis concerning the phago-lysosomal fusion in Figure 6 is very interesting. One aspect that is not entirely clear is how and if loading with BAPTA-AM would or could affect the Ca2+ content within the lysosomes or phagosomes. If STIM1 presence leads to activation of Orai on the phagosomal membranes as proposed by the authors in earlier work (Nunes et al 2012), deletion of STIM1 would likely increase phagosomal Ca2+ content, whereas BAPTA AM in the cytosol or in the lumen of the phagosome would decrease it.

The reviewer brings up a very interesting point, which we also discuss in our response to reviewer #3, point 8. Indeed, we predict that BAPTA loading and STIM1 deletion should impact the phagosomal Ca^{2+} content differently. We do not expect significant BAPTA buffering in the phagosome lumen, for two reasons. First, cells were pre-incubated in low concentrations of BAPTA-AM (40 µM) and the chelator was washed off before adding beads, thus the free BAPTA concentration in the lumen is negligible. Second, BAPTA buffers Ca^{2+} efficiently at nanomolar concentrations but has a negligible impact at concentration higher than 10 µM as it is fully saturated. In a previous publication, we exploited this property to study the kinetics of ER refilling in cells maintained in Ca^{2+}-free conditions, and could readily measure changes in the ER Ca^{2+} concentration in cells loaded with BAPTA-AM (Shen, JBC 2011 [14]). BAPTA is therefore not expected to impact the free Ca^{2+} concentration of organelles as long as it is maintained above micromolar levels. This is the case for lysosomes, and the same high concentration is expected in early phagosomes as the particles is ingested together with saline solutions of millimolar Ca^{2+} concentrations. The effects of BAPTA are thus mainly exerted through cytosolic buffering, via the suppression of Ca^{2+} microdomains around phagosomes and the increased Ca^{2+} buffering capacity conferred by the chelator. How these effects impact the phagosomal Ca^{2+} concentration depends on the Ca^{2+} regulatory properties of the Ca^{2+} channels, pumps, and exchangers mediating uptake and release and on the affinity of the uptake systems for Ca^{2+}. The outcome is difficult to predict, but we expect BAPTA to have a greater impact as it abrogates Ca^{2+} microdomains around phagosomes while STIM1 deletion has only a partial effect. Ca^{2+}-dependent fusion with other organelles should also be more impacted as discussed below.

Regarding the effects of STIM1 deletion, we agree with the reviewer that the deletion of STIM1 should increase phagosomal Ca^{2+} content, by preventing the release of Ca^{2+} across STIM1-gated channels. On the other hand, STIM1 deletion impairs the fusion of phagosomes with endosomes and lysosomes, Ca^{2+}-rich vesicles that might deliver an additional Ca^{2+} payload as well as Ca^{2+} pumps or exchangers that could mediate the refilling of phagosomes. The net outcome of these two processes is difficult to predict, but our observation that periphagosomal Ca^{2+} hotspots can be sustained for several minutes and are inhibited by internalized GSK suggest that the phagosomal Ca^{2+} concentration remains above micromolar levels. We attempted to measure the free luminal Ca^{2+} concentration within phagosomes, but the measurements are complicated by the limited availability of Ca^{2+} sensitive dyes that can be coupled to beads or loaded into phagosomes and by the pH sensitivity of BAPTA-based

probes, whose affinity for Ca^{2+} decreases by several orders of magnitudes at pH below 6. Our preliminary measurements are reported in the response to reviewer 1's point 10.

If and how STIM1 deletion affects lysosomal Ca^{2+} content is a different, very interesting question. Lysosomes make extensive contacts with the ER but STIM1 has so far not been involved in these interactions, the Ca^{2+} channels involved being of the TPC, TRPM and TRPML families [15]. Ca^{2+}-hydrogen exchangers have been proposed to maintain lysosomal calcium content higher than endosomal calcium, driven by the proton gradient generated by V-ATPases [16] but their molecular identity in higher mammals remains to be shown. Whether STIM1 participates in these contacts or can regulate lysosomal [Ca^{2+}] has not been investigated to date.

Also the % OVA degradation is significantly different only in the first 30-60 min whereas the P-L fusion Index is affected by STIM1 only at 90 min.

Another interesting point. Indeed, our OVA degradation assay reported that proteolysis was impaired at 30 and 60 min while a significant effect was only observed at 90 min in our phago-lysosome fusion assays. This suggests that proteases might be delivered early to DC phagosomes, via fusion with endosomes. To assess this possibility, we have measured endosome-phagosome fusion, using Alexa-594 dextran loaded into endosomes for only 15 min, instead of 3 hrs followed by an overnight chase. Interestingly we see an endosome fusion defect at 15 and 30 min after phagocytic ingestion, coinciding with the greatest difference in OVA degradation (Fig 7b, d). We further measured the activity of IRAP (Fig 7e), an endosomal enzyme critical for cross-presentation, and found that this activity was significantly decreased in STIM1 deficient cells. This suggests that endosomal proteases rather than lysosomal proteases are preferentially affected by the depletion of STIM1, consistent with the larger reduction in OVA degradation that we observed at early time points. We thank the reviewer for this insightful comment that prompted us to do these experiments. .

Even if STIM1 function is only structural, BAPTA AM would lead to an increased clustering and increased interaction sites between ER and phagosomes (Fig. 3). Analysis of the contact sites (as in Fig. 3) in the presence of BAPTA AM might clarify this.

As discussed in point 5) above, we do not predict that BAPTA-AM will induce SOCE as the chelator only accumulates to significant extents in the cytoplasm, and does not impact the free ER Ca^{2+} concentration. Indeed we have shown previously that ER Ca^{2+} refilling can still occur in BAPTA-AM loaded cells, presumably because the affinity of the SERCA pump for Ca^{2+} is higher than BAPTA's affinity for Ca^{2+} [14]. As BAPTA-AM can have pleiotropic effects by impacting on the activity of Ca^{2+} dependent cellular processes, we did not perform this experiment as we feared that the interpretation of the EM data will be ambiguous.

Minor:
Line 32: of which there are two paralogs (STIM1 and STIM2) each of which show additional splice variants

This is now mentioned in the introduction

Line 35: Please include references for the remodeling role of STIM proteins in ER junction

formation

This is now added to the introduction

Reviewer #3 (Remarks to the Author):

This manuscript reports that STIM1 is required for optimal dendritic cell (DC) migration, phagosomal maturation and, most importantly, for antigen cross-presentation. Using both in vivo and in vitro measurements, the authors report a marked diminution of antigen cross-presentation efficiency. They propose that impaired chemotaxis and antigen proteolysis in phagosomes account for these observations.

Despite the considerable number of sophisticated approaches applied, it was unclear to me what mechanism is proposed to account for the reduced cross presentation. On one hand, the authors report reduced contacts between the ER and phagosomes in cells lacking STIM1. They also find that calcium hotspots around the phagosome are reduced, which may account for the reduced fusion and maturation. These observations, however, generate a number of questions and issues that are critical and, in my view, have not been resolved in the current version. These include:

1) STIM1 associates with the plasma membrane when the luminal calcium of the ER is depleted, but is otherwise not attached to it. Do the authors envisage a similar process connecting the ER to phagosomes via STIM1? If so, why is STIM1 activated during phagosome maturation? Is the calcium in the ER depleted at the time when they analyzed the ER contacts (the time should be specified in the text and figure legend). If so, this should be demonstrated and the underlying mechanism should be defined. If not depleted of calcium, why would the ER associate with phagosomes in a STIM1-dependent manner? Is this a novel mode of function of STIM1 and, if so, is it unprecedented?

The reviewer brings up several interesting mechanistic questions regarding the generation and maintenance of STIM1-mediated ER-phagosomes contact sites. We do not postulate a new mode of STIM1 activation as there is ample evidence in the literature that ER Ca^{2+} release occurs during phagocytosis following activation of receptors coupled to PLC or PLD [17]. The ensuing decrease in the free ER Ca^{2+} concentration promotes the exposure of STIM1 cytosolic tail that binds to phosphoinositides and to ORAI1 and TRPC channels, enabling STIM1 to act as an ER tether. We have observed that contact sites occur even at cup stage by EM indicating that STIM1 may be recruited during early stages of receptor engagement. Ca^{2+} signalling activity is highest during the initiation of phagocytosis but phagosomes are more frequently observed 30 min later and we chose this time point for our EM analysis of ER contacts and for Ca^{2+} imaging. The time point is now specified in the legend of Figure 4. Our recordings of periphagosomal Ca^{2+} elevations establish that Ca^{2+} fluxes occur at the time selected to analyse ER contacts. We now demonstrate that Ca^{2+} release from the ER contribute to these local elevations as their occurrence is diminished following inhibition of the InsP3R with xestospongin (Fig 3c). We further show that the Ca^{2+} hotspots coincide with STIM1 clusters around phagosomes (Fig 3e). To prove that the ER is depleted locally near phagosomes would require subcellular imaging of the changes in the ER Ca^{2+} concentration with probes like D1ER or GEM-CEPIA, experiments that are challenging given the small size and dynamic nature of the ER-phagosomes contact sites. We did not attempt to perform such recordings as we believe that sufficient available evidence points to ER Ca^{2+} depletion

as the mechanism that initiate STIM1 recruitment to phagosomes, and that there is no need to postulate an alternative mechanism.

Whether STIM1 recruitment to phagosomes is static or dynamic is a more difficult question to answer. We previously reported that STIM1 clusters form, move, and dissipate dynamically around phagosomes [18], but it is unclear whether STIM1 populates and leaves stable contacts or whether it is dynamically generating new ones. On the other hand, we also observed that periphagosomal Ca^{2+} hotspots could persist for tens of minutes, an unusually long time for Ca^{2+} signals [18]. The increased avidity of the extended, active STIM1 for phosphoinositides could be a mechanism for retaining STIM1 at phagosomes, which are enriched in negative phosphoinositides at early stages of phagocytosis [19]. Alternatively, it is possible that similar to ER-mitochondria contacts, and in contrast to ER-PM contacts, ER-phagosome contacts might exclude SERCA pumps and that the ensuing lack of ER refilling would maintain STIM1 in an active configuration. ROS modification of STIM1 or differential binding with one of STIM1's many binding partners could also maintain contacts in place once recruited. Elucidating the retention mechanisms of STIM1 at phagosomes is an important question that will require an in-depth investigation which we are eager to take on but that, we believe, is outside of the scope of the present study.

2) If the mechanism of action of STIM1 involves calcium release from phagosomes, what activates STIM1 and, as importantly, what is the evidence that SOCE or TRPC channels exist and are functional in the maturing phagosome?

The mechanism of STIM1 activation is discussed in point 1 above. Regarding the channels involved, we previously showed that ORAI isoforms traffic to phagosomes in a dynamic manner when expressed in HL-60 cells, with Orai1 being present at cups and later depleted in maturing phagosomes, Orai2 being equally present at cups and internal phagosomes, and Orai3 being enriched in internal phagosomes as compared to cups. To address the reviewer's question, we have used a pharmacological approach to test whether ORAI1 channels are also present on DC phagosomes. We now show that the Orai1 inhibitor GSK7975A, applied during the ingestion of the particle to act from the intraphagosomal side, reduces the incidence of periphagosomal Ca^{2+} microdomains, measured at 30 min, by 30% in DC from WT mice but has no significant effect in cells lacking STIM1 (Fig 3c). This demonstrates that STIM1-gated ORAI1 channels are present and functional in mature phagosomes from dendritic cells. An even larger inhibition was observed following inhibition of the InsP3R with xestospongin (Fig 3c), indicating that Ca^{2+} release from the ER plays a dominant role in triggering the local elevations, likely by simultaneously fuelling cytosolic Ca^{2+} signals and by promoting the activation of STIM1 as discussed in point 1 above. Interestingly, Ca^{2+} microdomains were still observed after 90 min but were no longer STIM1-dependent, suggesting that different Ca^{2+} transporters or channels are engaged as phagosomes matures, consistent with our data in HL-60 cells.

Would the internal acidification be compatible with their activity? Late endosomes/lysosomes contain TRP-ML1 but, to my knowledge, this channel is not activated by STIM1.

Indeed ORAI channels are inhibited at external pH below 6 and activated at extracellular pH above 8 [20, 21]. The activity of ORAI channels is therefore expected to decrease gradually as phagosomes acidify, but also to increase as phagosomes alkalinize. Inhibition at acidic pH might explain the disappearance of STIM1-dependent Ca^{2+} hotspots at late time points, but the average pH values that we recorded 30 min post-phagocytosis are still compatible with

active ORAI channels. At these pH values (pH 7.3 and 6.6 with FITC and pHrodo, respectively, Fig 6c and 6d), the amplitude of the currents recorded in ORAI channels exogenously expressed in HEK cells are 10% (pH 7.3) to 50% (pH 6.6) lower than the currents recorded at pH 7.4 [21]. Moreover, we observed a bimodal distribution of phagosomal pH with two populations of acidic and alkaline phagosomes (Fig 6e). A significant fraction of phagosomes thus has a luminal pH that actually increases the activity of ORAI channels. The pH sensitivity of ORAI1 channels might therefore favour the generation of periphagosomal Ca^{2+} hotspots by increasing the activity of ORAI channels in alkaline phagosomes. We thank the reviewer for this insightful comment and now discuss this issue in our revised MS (p. 15).

TRPML1, TRPM4 and TRPM2 have been suggested to play a role in Ca2+ signalling in DCs during phagocytosis and were all suggested to play a role in lysosomes [22], but none of these channels is activated by STIM1. As discussed in point 2 above it is possible that these channels are recruited to late phagosomes and contribute to the Ca^{2+} release from phagosomes at later time points.

3) The protease content of phagosomes of WT and STIM1-KO cells was found to be indistinguishable. This seems incompatible with the finding that lysosome fusion is inhibited. The authors postulate that perhaps other proteases not investigated are responsible for the observed differences. However, differential delivery of another protease would require that the putative protease be selectively localized in a subpopulation of lysosomes. What is the evidence that this can occur?

We now show that the activity of the insulin-regulated aminopeptidase (IRAP) is decreased in STIM1-deficient phagosomes (Fig. 7e). IRAP is localized in endosomal storage compartments and IRAP deficiency decreases cross-presentation by 50% in human DC [23]. We further report that STIM1-deficient cells have an endosome fusion defect at 15 and 30 min after phagocytic ingestion (Fig 7d). This suggests that endosomal proteases rather than lysosomal proteases are preferentially affected by the depletion of STIM1, consistent with the larger reduction in OVA degradation that we observed at early time points, as also noted by reviewer #2.

4) In the end, it is unclear whether more proteolysis favors cross-presentation, or whether the opposite is true. The notion that reduced phagosomal acidification promotes cross-presentation as a result of reduced proteolysis seems incompatible with the interpretation of this paper.

We realize that our findings might at first glance challenge the concept that reduced proteolysis leads to increased cross-presentation. However, we would like to point out that this concept ignores the basic fact that peptides of 8-9 amino acids are what is loaded on MHC-I complexes. These peptides need to be generated from intact proteins, therefore some proteolysis must occur to allow cross-presentation. We agree that excessive proteolysis leaving only single amino-acids would impair cross-presentation, but disagree with the dogmatic view that preventing the activity of proteases in phagosomes will necessarily favour cross-presentation. Our data instead suggest that appropriate, finely regulated levels of proteolysis must be reached to favour cross-presentation. In this regard, the identification of IRAP as the protease impacted by STIM1 deletion is significant, as the activity of this peptidase is coupled to MHC-I loading. IRAP inhibition might influence cross presentation by uncoupling this proteolysis step from the peptide loading process. We therefore believe that our findings provide a significant conceptual advance to the field by showing the limits

of the current dogma and by highlighting specific Ca^{2+} regulated processes that are required for efficient cross-presentation. We have expanded our discussion to clarify this point.

5) Along the same lines: is the very acidic pH reported in the majority of the phagosomes consistent with the results of Amigorena's group?

At the population level, our results are most consistent with the findings of Yates and Russell reporting that DC phagosomes acidify [24], although the acidification was slower and more limited in our case. They do not, however, refute earlier findings by the Amigorena's group reporting an alkalinisation, as a significant population of our phagosomes were very alkaline, even at late time points (Fig. 5e). By measuring single phagosomes on the microscope, we achieve here a level of resolution superior to these previous measurements in populations of cells by flow cytometry or plate readers. This enabled us to document the pH heterogeneity of DC phagosomes, which exhibit a bimodal distribution with two populations of acidic and alkaline phagosomes (Fig 6e). We previously reported a similar heterogeneity in the phagosomal pH of neutrophils ingesting zymosan particles [8], and showed that the alkalinisation of neutrophil phagosomes depends on the amount of ROS produced by the NADPH oxidase, as initially reported by the Amigorena's group [9]. The level of ROS produced is thus a key determinant of the phagosomal pH, and both ROS and pH are regulated at the level of single phagosomes. This greatly complicates the comparison of different studies, as differences in the levels of ROS produced due to different culturing conditions or genetic variation in mouse strains in the different labs might alter the proportion of acidic vs. alkaline phagosomes. Future studies aiming to clarify the mechanisms that control the phagosomal pH in DC should take into account the fluctuations that occur at the level of single phagosomes.

Is it compatible with effective cross-presentation?

We believe it is. We observed a bimodal distribution of phagosomal pH not only at the population level, but also within the same cell (Fig 6f). Peptides could therefore be generated in one phagosome and destroyed in another, allowing the same cell to present some peptides efficiently and to destroy others. The BMDCs used in our study and in the companion paper were able to cross-present sufficiently to decrease tumor growth in the adoptive transfer model employed by the Manoury group.

Along the same lines, the companion manuscript of Manoury et al does report changes in phagosomal pH, in contrast to the result in the present paper. What account for this apparent discrepancy? Does this imply that UNC93 is having effects other than through STIM1?

Exactly. The UNC93B1 3d mutation phenotype has a stronger effect on cross-presentation than STIM1 knockout in vitro, implying additional defects beyond the regulation of STIM1. One of these effects is indeed an effect on phagosomal pH.

6) The large difference between the pH values reported using two different methods needs to resolved. Which one is right?

This difference likely derives from the widespread non-Gaussian distribution of the pH of DC phagosomes, which ranges from pH 4 to pH 9 (Fig 6e). In these conditions, the average pH is a parameter that depends heavily on the pKa of the pH probe used and on the proportion of acidic and alkaline phagosomes in the population recorded. The reason for this is that a given

probe will accurately report the pH of phagosomes only when this value is close to the probe's pKa and will provide meaningless information when the pH deviates by more than one 1 pH unit from its pKa. When selecting a pH probe, it is therefore important to choose one whose pKa matches the pH of the compartment measured, which is simply impossible in this case given the widespread pH distribution of DC phagosomes. We initially used FITC to allow the comparison of our pH data with earlier studies using fluorescein-based measurements. The presence of a large population of acidic phagosomes whose pH could not be resolved made us switch to pHrodo, whose acidic pKa allows a better estimate of the pH of this population. However, pHrodo cannot resolve the pH of alkaline phagosomes and underestimates the contribution of this population. In our attempt at measuring phagosomal calcium levels we measured phagosomal pH using a combination of FITC and Oregon Green, a probe with a lower pKa, but identical pH-sensitive wavelengths as FITC. Due to the technical challenges facing this complex analysis we have opted not include these data in the new manuscript, as discussed above in our answer to reviewer 1's point 10. We have pasted these data below for the reviewer's benefit. They confirm the pHrodo data, which provides a more accurate measure due to its lower pKa and increased dynamic range at acidic pH, now emphasized in the discussion, and suggest that the majority of DC phagosomes are indeed acidic.

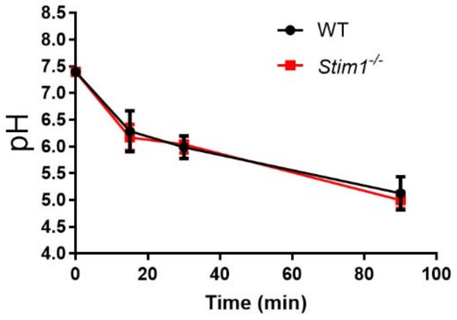

Fig 2. Average phagosomal pH measured with OVAb coupled to lysine fixable FITC-dextran and Oregon Green-dextran.

7) Were similar concentrations of chemoattractant used to measure calcium and chemotaxis? If not, the conclusions need to be tempered down accordingly.

Yes. This was mentioned in the Mat and Methods and is now more clearly specified

8) What is the evidence that the calcium hotspots originate from the phagosome? If that is not the correct interpretation, what is their source (ER? Lysosomes?) and their relevance to the fusion process? Are these sustained events and, if so, why is the phagosomal calcium not soon depleted?

Please see our response to point #2 above and the discussion for reviewer #2, point 5, regarding the source of Ca^{2+} hotspots and the impact of our manipulations on phagosomal Ca^{2+} concentration. The hotspots can be sustained for several minutes, and there is ample evidence in the literature supporting a role for Ca^{2+} in promoting endolysosomal fusion events [25]. Our pharmacological data (Fig 3c) indicate that Ca^{2+} release from the ER and from phagosomes both contribute to these Ca^{2+} hotspots, consistent with our previous findings [18, 26]. Ca^{2+} release from the ER can be sustained as the ER can be continuously refilled by SERCA locally or elsewhere within the network, but to account for persistent Ca^{2+} hotspots such a mechanism might require a continuous local production of InsP3 to induce the release

of Ca^{2+} from vicinal ER stores. Alternatively Ca^{2+}-induced Ca^{2+} release might play a role, as well as facilitation of InsP3R activation by junctate[26]. Why the phagosome is not depleted is a good question. Delivery of Ca^{2+} pumps or exchangers could mediate the refilling of phagosomes and prevent their depletion, and fusion with lysosomes could deliver additional Ca^{2+} payloads. We have attempted to measure the phagosomal Ca^{2+} with dyes, but the measurements are complicated by the limited availability of Ca^{2+} sensitive dyes that can be coupled to beads or loaded into phagosomes and by the pH sensitivity of BAPTA-based probes, whose affinity for Ca^{2+} decreases by several orders of magnitudes at pH below 6.

[Redacted].

[Redacted]

9) What is the relevance of the inhibitory effect of DPI and of concanamycin A to calcium?

We used DPI and ConcA as positive controls to validate our assays, as these compounds inhibit cross-presentation by disrupting phagosomal ROS and pH homeostasis. We now show that the ORAI1 channel inhibitor GSK7975A prevents phago-endosome fusion (Fig 6e), further confirming that Ca^{2+} signals contribute to the cellular events associated with cross-presentation.

10) In Figure 2 the authors quantify calcium transients. However these presumably reflect mostly calcium release from the ER. How are they relevant to STIM function?

Ca^{2+} responses evoked by chemoattractants comprise two components, an initial rapid elevation caused by the release of Ca^{2+} from the ER and a delayed elevation reflecting the entry of Ca^{2+} across membrane channels. A clearly biphasic response is evoked by fMIFL and PAF (Figs 2b and 3b). The lack of the delayed elevation is indicative of the defect in SOCE imparted by the ablation of the *Stim1* gene. Comparison of the area under the curve of the evoked responses, a parameter that integrates the release and influx components, indicates that STIM1-mediated influx contributes ~50% of the response evoked by fMIFL and SDF1. We tested SDF-1 and CCL21 because these chemokines are known to be involved in DC honing and were suggested to elicit Ca^{2+} signals upon activation.

11) Is CCL21 not more relevant to cross-presentation in situ than is fMIFL? If so, is the explanation offered adequate?

CCL21 is indeed one of the most important chemoattactrants for DC honing to lymph nodes, but ablation of the CCL21 receptor CCR7 does not completely abolish DC honing, indicating that other signals contribute. The SDF-1 receptor CXCR4 expressed in lymphatic vessels also contributes to migration of cutaneous DCs to lymph nodes upon activation [27] and thus may represent an earlier, short-range signal. fMIFL is a chemotactic bacterial peptide that attracts innate immune cells to the site of infection and we used it to test the ability of cells to be recruited to the site of infection. Thus, the defects we expect based on our calcium data fit: Attraction to the site and migration from the site, both early events, are partially (though not completely) impaired at early time points, and non-calcium dependent signals would still attract DCs. If the DC migrate in a non-directional manner after phagocytosing it might eventually sense the CCL21 gradient emanating from the lymph node, and hone back, albeit in a delayed manner. This is what we believe is happening, and have added to the discussion to clarify.

12) Why does OVAb stimulate the NADPH oxidase?

Ovalbumin engages the mannose receptor [28] which is coupled to the Syk kinase, and in macrophages MR ligation is coupled to p47phox and arachidonic acid-NADPH oxidase signaling pathways [29]. In addition, nearly all phagocytic events trigger integrin receptor signalling. Integrin signalling is known to activate PLC, PLD and PKC pathways. These will cause the phosphorylation of the cytosolic p47phox subunit and promote its translocation to membranes bearing the catalytic gp91phox subunit, thereby activating the NADPH oxidase.

13) Is cell-associated bead fluorescence an adequate measure of phagocytosis? Are there no adherent beads attached extracellularly or have they been ruled out? Please explain.

We tried to quench the fluorescence of non-phagocytosed beads with trypan blue, but saw little difference with our standard protocol in flow cytometry experiments. Our procedure includes several washing steps in EDTA-containing buffer, which we believe dissociates most of the cell-attached beads. Our cytometry data are concordant with our microscopy based quantification of phagocytosis, where adherent beads can be clearly distinguished, and with the report by Vaeth et al [30]. Therefore we are confident that our flow cytometry results are an adequate measure of phagocytosis.

References

1. Dixit N, Yamayoshi I, Nazarian A, Simon SI. Migrational guidance of neutrophils is mechanotransduced via high-affinity LFA-1 and calcium flux. *Journal of immunology* **187**, 472-481 (2011).

2. Shao Z, Gaurav R, Agrawal DK. Intermediate-conductance calcium-activated potassium channel KCa3.1 and chloride channel modulate chemokine ligand (CCL19/CCL21)-induced migration of dendritic cells. *Transl Res* **166**, 89-102 (2015).

3. Scandella E*, et al.* CCL19/CCL21-triggered signal transduction and migration of dendritic cells requires prostaglandin E2. *Blood* **103**, 1595-1601 (2004).

4. Giacomello M*, et al.* Ca2+ hot spots on the mitochondrial surface are generated by Ca2+ mobilization from stores, but not by activation of store-operated Ca2+ channels. *Mol Cell* **38**, 280-290 (2010).

5. Christensen KA, Myers JT, Swanson JA. pH-dependent regulation of lysosomal calcium in macrophages. *Journal of cell science* **115**, 599-607 (2002).

6. Poburko D, Santo-Domingo J, Demaurex N. Dynamic regulation of the mitochondrial proton gradient during cytosolic calcium elevations. *The Journal of biological chemistry* **286**, 11672-11684 (2011).

7. Tlili A, Dupre-Crochet S, Erard M, Nusse O. Kinetic analysis of phagosomal production of reactive oxygen species. *Free Radic Biol Med* **50**, 438-447 (2011).

8. El Chemaly A, Nunes P, Jimaja W, Castelbou C, Demaurex N. Hv1 proton channels differentially regulate the pH of neutrophil and macrophage phagosomes by sustaining the production of phagosomal ROS that inhibit the delivery of vacuolar ATPases. *Journal of leukocyte biology* **95**, 827-839 (2014).

9. Savina A*, et al.* NOX2 controls phagosomal pH to regulate antigen processing during crosspresentation by dendritic cells. *Cell* **126**, 205-218 (2006).

10. Zhang H*, et al.* STIM1 calcium sensor is required for activation of the phagocyte oxidase during inflammation and host defense. *Blood* **123**, 2238-2249 (2014).

11. Sallusto F, Lanzavecchia A. Efficient presentation of soluble antigen by cultured human dendritic cells is maintained by granulocyte/macrophage colony-stimulating factor plus interleukin 4 and downregulated by tumor necrosis factor alpha. *The Journal of experimental medicine* **179**, 1109-1118 (1994).

12. Platt CD*, et al.* Mature dendritic cells use endocytic receptors to capture and present antigens. *Proceedings of the National Academy of Sciences of the United States of America* **107**, 4287-4292 (2010).

13. Alloatti A*, et al.* Toll-like Receptor 4 Engagement on Dendritic Cells Restrains Phago-Lysosome Fusion and Promotes Cross-Presentation of Antigens. *Immunity* **43**, 1087-1100 (2015).

14. Shen WW, Frieden M, Demaurex N. Local cytosolic Ca2+ elevations are required for stromal interaction molecule 1 (STIM1) de-oligomerization and termination of store-operated Ca2+ entry. *The Journal of biological chemistry* **286**, 36448-36459 (2011).

15. Patel S. Function and dysfunction of two-pore channels. *Sci Signal* **8**, re7 (2015).

16. Penny CJ, Kilpatrick BS, Eden ER, Patel S. Coupling acidic organelles with the ER through Ca(2)(+) microdomains at membrane contact sites. *Cell calcium* **58**, 387-396 (2015).

17. Nunes P, Demaurex N. The role of calcium signaling in phagocytosis. *Journal of leukocyte biology* **88**, 57-68 (2010).

18. Nunes P, *et al.* STIM1 juxtaposes ER to phagosomes, generating Ca(2)(+) hotspots that boost phagocytosis. *Current biology : CB* **22**, 1990-1997 (2012).

19. Bohdanowicz M, Grinstein S. Role of phospholipids in endocytosis, phagocytosis, and macropinocytosis. *Physiological reviews* **93**, (2013).

20. Beck A, Fleig A, Penner R, Peinelt C. Regulation of endogenous and heterologous Ca(2)(+) release-activated Ca(2)(+) currents by pH. *Cell calcium* **56**, 235-243 (2014).

21. Tsujikawa H, *et al.* Identification of key amino acid residues responsible for internal and external pH sensitivity of Orai1/STIM1 channels. *Sci Rep* **5**, 16747 (2015).

22. Patel S, Cai X. Evolution of acidic Ca(2)(+) stores and their resident Ca(2)(+)-permeable channels. *Cell calcium* **57**, 222-230 (2015).

23. Saveanu L, *et al.* IRAP identifies an endosomal compartment required for MHC class I cross-presentation. *Science (New York, NY)* **325**, 213-217 (2009).

24. Rybicka JM, Balce DR, Chaudhuri S, Allan ER, Yates RM. Phagosomal proteolysis in dendritic cells is modulated by NADPH oxidase in a pH-independent manner. *The EMBO journal* **31**, 932-944 (2012).

25. Kiselyov KK, Ahuja M, Rybalchenko V, Patel S, Muallem S. The intracellular Ca(2)(+) channels of membrane traffic. *Channels (Austin)* **6**, 344-351 (2012).

26. Guido D, Demaurex N, Nunes P. Junctate boosts phagocytosis by recruiting endoplasmic reticulum Ca2+ stores near phagosomes. *Journal of cell science* **128**, 4074-4082 (2015).

27. Kabashima K, *et al.* CXCL12-CXCR4 engagement is required for migration of cutaneous dendritic cells. *Am J Pathol* **171**, 1249-1257 (2007).

28. Burgdorf S, Lukacs-Kornek V, Kurts C. The mannose receptor mediates uptake of soluble but not of cell-associated antigen for cross-presentation. *Journal of immunology* **176**, 6770-6776 (2006).

29. Lefevre L, *et al.* The C-type lectin receptors dectin-1, MR, and SIGNR3 contribute both positively and negatively to the macrophage response to Leishmania infantum. *Immunity* **38**, 1038-1049 (2013).

30. Vaeth M, *et al.* Ca2+ Signaling but Not Store-Operated Ca2+ Entry Is Required for the Function of Macrophages and Dendritic Cells. *Journal of immunology* **195**, 1202-1217 (2015).

Reviewers' comments:

Reviewer #1 (Remarks to the Author):

NCOMMS-16-24040 answer to the rebuttal letter

This article aims to demonstrate the role of STIM-1 in the migration, phagosomal maturation and cross -presentation in the murine dendritic cells (DCs). In fact this latter skill has a huge importance in the therapeutic strategy against cancer using DCs.

My first review of this manuscript drove me to ask several questions concerning the involvement of STIM1 in these different skills of murine DCs. My main question was about the demonstration of STIM1 involvement into the cross presentation in murine DCs. The indirect clues presented, i.e. local Ca2+ defects, close to STIM1 molecule and the defect of cross represented a gap that the presented experimental results could not cross. The authors have deeply changed the article by adding both experiments and arguments and the conclusions they gave better matched with the experimental results. The aim of the paper have been reduced to what it can be really demonstrated. May be, should the author indicate that the results relate to murine DCs only?

My comments on the text have been taken into account and the several changes work for me. Nevertheless, underlined changes would have helped us to check the changes. There are still typographical misprints in the first page.

To my opinion, the authors have really increased the scientific quality and the rigorous of the interpretation of their experimental results. This article must be accepted for publication.

Dr. Florence VELGE-ROUSSEL
Team EA 4245 "Dendritic cells,Immunomodulation and Grafts "
University F. Rabelais of Tours, France
Chair of Cost Action BM1406

Reviewer #2 (Remarks to the Author):

In their revised version the authors have satisfactorily addressed my concerns.

Reviewer #3 (Remarks to the Author):

The revised version of this manuscript is much improved and the authors should be congratulated for their willingness to undertake additional experiments. There are some remaining questions, some of which arise from the new data included in this revision.

1) Does cleavage of Leu-AMC detect only phagosome-associated IRAP, or total cellular IRAP activity? If, as I suspect, the latter is being measured in Figure 7e, why is it different in the Stim1 knockouts? And what is the relationship between this observation and the fusion of endosomes (or lack thereof) reported in Figure 7c-d?
2) How do the authors know (or why do they assume) that their FRET assay is a suitable reporter of fusion of the IRAP-containing compartment with phagosomes?
3) Can the authors provide a plausible explanation for the finding that the phagosomal pH distribution is bimodal? What underlies this distribution?
4) Does xestospongin prevent the accumulation of STIM1 around the phagosome?

The reviewer's comments are in plain letters, and *our responses in italics*

Reviewer #1 (Remarks to the Author):

This article aims to demonstrate the role of STIM-1 in the migration, phagosomal maturation and cross -presentation in the murine dendritic cells (DCs). In fact this latter skill has a huge importance in the therapeutic strategy against cancer using DCs.

My first review of this manuscript drove me to ask several questions concerning the involvement of STIM1 in these different skills of murine DCs. My main question was about the demonstration of STIM1 involvement into the cross presentation in murine DCs. The indirect clues presented, i.e. local Ca2+ defects, close to STIM1 molecule and the defect of cross represented a gap that the presented experimental results could not cross. The authors have deeply changed the article by adding both experiments and arguments and the conclusions they gave better matched with the experimental results. The aim of the paper have been reduced to what it can be really demonstrated. May be, should the author indicate that the results relate to murine DCs only?

That the results relate to murine DCs is now directly mentioned in the abstract

My comments on the text have been taken into account and the several changes work for me. Nevertheless, underlined changes would have helped us to check the changes. There are still typographical misprints in the first page.

We apologize for the oversight, and have now carefully rechecked the first page

To my opinion, the authors have really increased the scientific quality and the rigorous of the interpretation of their experimental results. This article must be accepted for publication.

We thank the reviewer for their efforts and the acceptance of our manuscript.

Reviewer #2 (Remarks to the Author):

In their revised version the authors have satisfactorily addressed my concerns.

We thank the reviewer for their efforts and acceptance of our manuscript.

Reviewer #3 (Remarks to the Author):

The revised version of this manuscript is much improved and the authors should be congratulated for their willingness to undertake additional experiments.

We thank the reviewer for their insightful comments which helped us improve our manuscript.
There are some remaining questions, some of which arise from the new data included in this revision.

1) Does cleavage of Leu-AMC detect only phagosome-associated IRAP, or total cellular IRAP activity? If, as I suspect, the latter is being measured in Figure 7e, why is it different in the Stim1 knockouts? And what is the relationship between this observation and the fusion of endosomes (or lack thereof) reported in Figure 7c-d?

The reviewer brings up a good point. We originally measured IRAP activity following immunoprecipitation of whole cell lysates with an anti-IRAP antibody to isolate IRAP activity from other leucyl aminopeptidases, but now realize that this approach was inappropriate as it does not report phagosome-associated activity. We have now performed the Leu-AMC cleavage assay on isolated phagosomes (without IP) and find a ~40% loss of activity in knockout phagosomes while the activity was preserved in whole-cell lysates (new Figure 7e). These data indicate that IRAP activity is indeed reduced in phagosomes and interestingly also in immuno-precipitates from whole cell lysates, despite similar levels of IRAP being detected by western blot in these lysates. We speculate that the latter defect might reflect defective endosomal or macropinosomal maturation in STIM1-depleted cells, perhaps through decreased formation of ER-endosome contact sites. To document a putative ER-endosome defect would require a much more in depth study. To avoid ambiguities, we have removed the IP data and conservatively conclude that that leucyl aminopeptidase activity is specifically decreased in isolated phagosomes, consistent with either a defect in the delivery of enzymes such as IRAP to phagosomes or with a loss of activity of these enzymes within phagosomes. In addition, we now show a ~20% reduction in periphagosomal IRAP immunostaining in STIM1 deficient cells (new Figure 7f), consistent with a failed delivery/decreased fusion mechanism. Together with our new data showing that dextran and IRAP labelling partially overlap (see below), we believe that these observations greatly strengthen the concept that reduced delivery of IRAP to phagosomes contributes to the defect in cross-presentation in STIM1 deficient cells. We do not exclude, however, the possibility that other leucyl aminopeptidases or that reduced activity within phagosomes may also contribute.

2) How do the authors know (or why do they assume) that their FRET assay is a suitable reporter of fusion of the IRAP-containing compartment with phagosomes?

The IRAP-containing compartment has been previously characterized to be a Rab7+, Stx6+early and late endosomal (but not lysosomal) compartment that co-localizes with soluble internalized ovalbumin [1]. Dextran internalization is assumed to occur mostly non-specifically, as a fluid-phase endocytic marker. However it has be shown that dextran internalization also occurs at least partly via the mannose receptor[2], which is the same receptor that recognizes ovalbumin[3].Thus it stands to reason that IRAP-containing endosomes would at least partly overlap with our dextran-labelled endosomal population. We have now performed immunofluorescence to determine whether colocalization between IRAP and fluorescently labelled dextran occurs. A subpopulation of dextran-labelled endosomes was positive for IRAP (new Supplementary Figure 7e), confirming our previous assumption. It should be noted however that these immunostainings were quite difficult because even using lysine-fixable forms of dextran and very small amounts of detergent, the dextran survives only very poorly the permeabilization step required to get a decent signal on the IF. Thus, while we can find cells with a dextran signal resembling the staining observed before permeabilization, much of the dextran signal was lost. For this reason we prefer not to do a quantification of the percent overlap between IRAP and dextran, as we do not believe that such a figure would be a representative estimate of the real percent overlap.

3) Can the authors provide a plausible explanation for the finding that the phagosomal pH distribution is bimodal? What underlies this distribution?

We believe this bimodal distribution is related to the heterogeneous production of ROS in individual phagosomes. We and others previously showed that the phagosomal pH and redox state are intimately linked and regulated at the single phagosome level in neutrophils [4, 5, 6]. Genetic and pharmacological data indicate that phagosomal ROS production inhibits the delivery of V-ATPase to phagosomes [7]. Phagosomes that produce low amounts of ROS are enriched in V-ATPase and rapidly acidify. The mechanisms by which this inhibition occurs remain unknown, but such a "switch" type of behaviour could lead to a bimodal distribution, as the result of the existence of two bistable states: one a ROS producing alkaline/neutral phagosome, and the second an acidic V-ATPase containing phagosome (see [8] for a discussion on bistability and bimodal distributions).In neutrophils, decreasing the production of ROS genetically or pharmacologically resulted in a bimodal distribution of phagosomal pH [4]. We speculate that a similar situation may occur naturally in DCs, which produce much lower levels of ROS than neutrophils. We have expanded our discussion slightly to include these thoughts.

4) Does xestospongin prevent the accumulation of STIM1 around the phagosome?

Due to the difficulty in transfecting DCs we did not test whether this occurs or not in DCs, however in looking at images from our previous study conducted in mouse fibroblasts [9] we do still see examples of STIM1 accumulation near phagosomes even in the presence of xestospongin (an example is pasted below). It should be noted that since xesto strongly inhibits phagocytosis we only apply it after cells have been allowed to internalize beads for 20 min – thus the periphagosomal STIM1 we observed may have already been recruited during the initial period of phagocytosis. Without formally quantifying, our feeling however is that STIM1 recruitment levels are reduced upon xesto treatment, and we have now made a note in the results section that loss of STIM1 accumulation at contact sites may contribute to the reduced periphagosomal Ca^{2+} signalling in the presence of xestospongin.

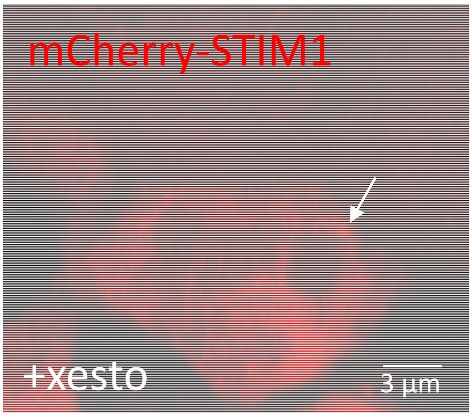

References

1. Weimershaus M, Maschalidi S, Sepulveda F, Manoury B, van Endert P, Saveanu L. Conventional dendritic cells require IRAP-Rab14 endosomes for efficient cross-presentation. *Journal of immunology* **188**, 1840-1846 (2012).

2. Sallusto F, Cella M, Danieli C, Lanzavecchia A. Dendritic cells use macropinocytosis and the mannose receptor to concentrate macromolecules in the major

histocompatibility complex class II compartment: downregulation by cytokines and bacterial products. *The Journal of experimental medicine* **182**, 389-400 (1995).

3. Burgdorf S, Lukacs-Kornek V, Kurts C. The mannose receptor mediates uptake of soluble but not of cell-associated antigen for cross-presentation. *Journal of immunology* **176**, 6770-6776 (2006).

4. El Chemaly A, Nunes P, Jimaja W, Castelbou C, Demaurex N. Hv1 proton channels differentially regulate the pH of neutrophil and macrophage phagosomes by sustaining the production of phagosomal ROS that inhibit the delivery of vacuolar ATPases. *Journal of leukocyte biology* **95**, 827-839 (2014).

5. Jankowski A, Scott CC, Grinstein S. Determinants of the phagosomal pH in neutrophils. *The Journal of biological chemistry* **277**, 6059-6066 (2002).

6. Levine AP, Duchen MR, de Villiers S, Rich PR, Segal AW. Alkalinity of neutrophil phagocytic vacuoles is modulated by HVCN1 and has consequences for myeloperoxidase activity. *PloS one* **10**, e0125906 (2015).

7. Nunes P, Demaurex N, Dinauer MC. Regulation of the NADPH oxidase and associated ion fluxes during phagocytosis. *Traffic* **14**, 1118-1131 (2013).

8. Mitrophanov AY, Groisman EA. Positive feedback in cellular control systems. *Bioessays* **30**, 542-555 (2008).

9. Guido D, Demaurex N, Nunes P. Junctate boosts phagocytosis by recruiting endoplasmic reticulum Ca2+ stores near phagosomes. *Journal of cell science* **128**, 4074-4082 (2015).

REVIEWERS' COMMENTS:

Reviewer #3 (Remarks to the Author):

The authors have adequately addressed my remaining concerns and, as far as I am concerned, the manuscript is now acceptable.
