# Supplementary material for: STIM1 promotes migration, phagosomal maturation and antigen cross-presentation in dendritic cells
Source: Nat Commun. 2017 Nov 24;8:1852. doi: 10.1038/s41467-017-01600-6 (PMC5701258; doi:10.1038/s41467-017-01600-6)
Supplement: Supplementary file 2 — Peer Review File [file 41467_2017_1600_MOESM2_ESM.pdf]

Description of Additional Supplementary Files

File Name: Supplementary Movie 1
Description: 3D Reconstruction of ER-phagosome membrane contact sites. The movie shows a FIB-SEM stack of a phagosome (the large white object) of a WT BMDC exposed to OVA-coated beads. The ER can be discerned as darkly staining long and flat membranes. Multiple sites of contact (MCS) between the ER and the phagosome are observed. The 3D reconstruction shows the phagosomal membrane in purple, the ER is partially reconstructed in light blue and MCS are highlighted in yellow.
